# Relative patterns of sexual activity and fertility among HIV positive and negative women—Evidence from 46 DHS

**DOI:** 10.1371/journal.pone.0204584

**Published:** 2018-10-17

**Authors:** Milly Marston, Basia Zaba, Jeffrey W. Eaton

**Affiliations:** 1 London School of Hygiene and Tropical Medicine, London, United Kingdom; 2 Department of Infectious Disease Epidemiology, Imperial College London, London, United Kingdom; Tulane University School of Public Health and Tropical Medicine, UNITED STATES

## Abstract

**Objectives:**

Projections of fertility of HIV positive women as ART scales up are needed to plan prevention of mother-to-child transmission (PMTCT) services. We describe differences in exposure to pregnancy between HIV positive and HIV negative women by age, region and national ART coverage to evaluate the extent to which behavioural differences explain lower fertility among HIV positive women and assess whether exposure to pregnancy has changed with antiretroviral treatment (ART) scale-up.

**Methods:**

We analysed 46 nationally representative household surveys in sub-Saharan Africa conducted between 2003 and 2015 to estimate risk of exposure to recent sex and pregnancy of HIV positive and HIV negative women by age using a log binomial model. We tested for regional and urban/rural differences and associations with national ART coverage. We estimated an adjusted fertility rate ratio of HIV positive to HIV negative women adjusting for differences in exposure to pregnancy.

**Results:**

Exposure to pregnancy differs significantly between HIV positive and negative women by age, modified by region. Younger HIV positive women have a higher exposure to pregnancy than HIV negative women and the opposite is true at older ages. The switch occurs at 25–29 for rural women and 30–34 for urban women. There was no evidence that exposure to pregnancy of HIV positive women have changed as national ART coverage increased. The inferred rate of fecundity of HIV positive women when adjusted for differences in exposure to pregnancy were lower than unadjusted fertility rate ratios in women aged 20–29 and 20–24 in urban and rural areas respectively varying between 0.6 and 0.9 over regions.

**Discussion:**

The direct effects of HIV on fertility are broadly similar across ages, while the dramatic age gradient that has frequently been observed is largely attributable to variation in relative sexual exposure by age.

## Background

Numerous studies have demonstrated that the relationship between HIV and fertility varies with age. Among the youngest women aged 15–19 years, fertility is higher among HIV positive women, while above age 25 the fertility of HIV positive women becomes increasingly lower than that of their HIV negative counterparts, termed ‘HIV associated subfertility [1–3]. Population based studies have also identified differences in HIV subfertility by region [3,4], urban and rural area [3] and speculated whether changes are associated with increased antiretroviral treatment (ART) roll out [3, 5, 6].

Accurate short-term projections for the number of HIV positive women are important for HIV surveillance and policy, for example to plan local provision of prevention of mother-to-child transmission (PMTCT) services and interpret HIV surveillance data for pregnant women to infer wider epidemic trends. Beyond documenting the empirical relationships between HIV and fertility, such projections require characterization of the mechanisms that explain the complex relationship between HIV and fertility in order to predict how this will change as the epidemic context evolves, in particular with the rapid scale-up of ART and changes in eligibility policy. The Spectrum model supported by UNAIDS currently assumes that the fertility of women on ART for longer than six months is the same fertility as HIV-negative women of the same age. A number of cohort studies have reported high rates of conception among women on long-term treatment [7,8]. However, direct comparisons with fertility of HIV-negative women in the same population are not readily available. A recent systematic review concluded that evidence was scant, but suggested lower fertility in HIV positive women on ART [9]. Marston et al estimated that with national ART coverage at high levels, the gap between fertility of HIV positive and HIV negative women has narrowed, but that fertility of HIV positive women remained lower than would be expected if HIV positive women on ART had the same fertility as HIV negative women [3].

Biological and socio-behavioural explanations have been hypothesised to explain HIV subfertility, and, as ART roll out increases, both causes of HIV subfertility could be affected. The physiological and immunological effects of HIV on reducing fertility could be attenuated if ART lessens the severity of women’s HIV disease. Reduced widowhood and divorce together with increased sexual activity due to improved health could increase exposure to pregnancy for HIV positive women compared to the pre-ART era.

It is well documented that at younger ages, age at sexual debut largely explains the relatively higher fertility in HIV positive women compared to their HIV negative counterparts [1, 2, 4, 10]. However, at older ages the contribution of differences in sexual behaviour to lower fertility among HIV positive women has been less thoroughly analysed. Sexual intercourse has been reported as less frequent in HIV positive women compared to HIV negative women [11, 12] but also modern contraceptive use is generally lower [12, 13]. The proximate determinants of fertility through which behavioural factors must act are (i) sexual intercourse and (ii) non-use of contraception. Therefore, quantifying the differences between HIV positive and HIV negative women’s exposure to sex without the use of contraceptives and a comparison of the ensuing pregnancy rates sheds light on the contribution of sexual behaviour to HIV subfertility, and may explain some regional differences in HIV subfertility and predict how this could change in the era of ART.

We use 46 nationally representative surveys from sub-Saharan Africa to estimate levels and trends of exposure to sex and to pregnancy outcome comparing HIV positive women to HIV negative women by region, place of residence and national ART coverage to assess how much of the HIV subfertility seen in these populations could be directly due to sexual behaviour.

## Methods

**Fig 1:**
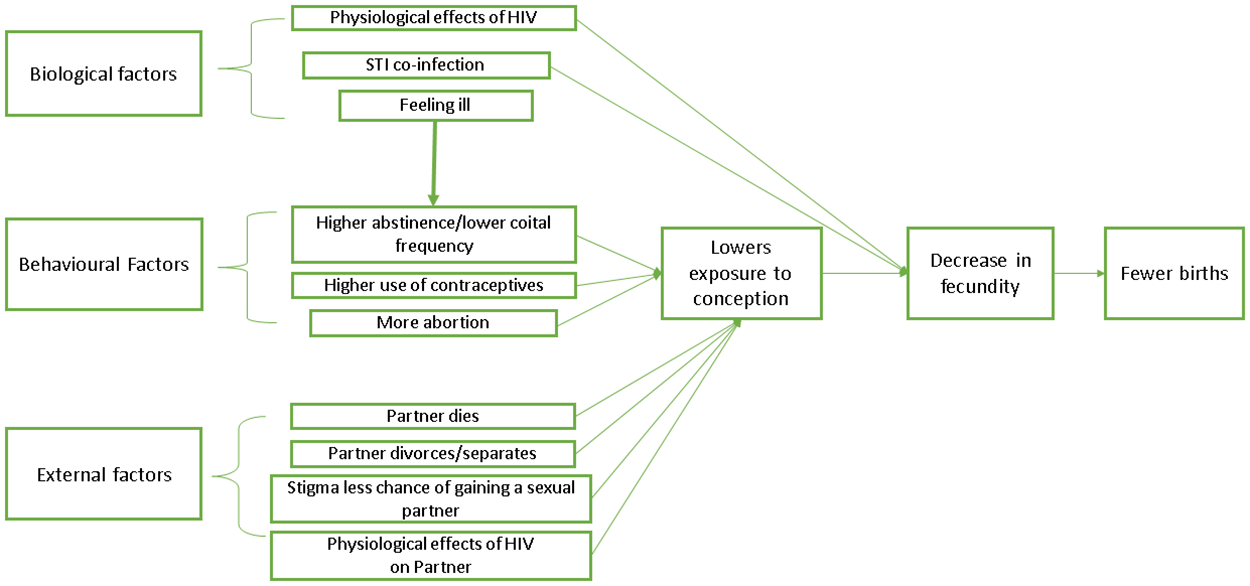
Conceptual framework for possible pathways explaining lower fertility in HIV positive women.

Fig 1 describes a conceptual framework for the possible effects of HIV on fertility. In this analysis, we aim to estimate the extent to which lower fertility is attributable to lower exposure to pregnancy due to behaviour modification associated with HIV, compared to biological factors decreasing fecundity which could potentially be directly ameliorated by successful ART. The ideal study to evaluate this would be to analyse individual-level conception rates during periods of exposure to pregnancy for HIV positive and HIV negative women. This is not possible in survey data because sexual activity and contraceptive use are only measured at the time of the survey, not over the duration of exposure to pregnancy.

Instead, to assess the contribution of differences in sexual behaviour (coital frequency and contraceptive use) to lower fertility in HIV positive women, we evaluate the relationship between age-specific exposure to recent sex (defined as sex in the last four weeks) and age-specific fertility rates of HIV positive women compared to HIV negative women. We hypothesise that, at the population level, exposure to recent sex is a measure of direct behavioural actions that lead to the possibility of a pregnancy. Therefore, if behavioural differences were the only factor determining differences in fertility, we would expect the relative rate of recent sex by HIV status to be the same as the relative fertility rates. On the other hand, if biological effects of HIV completely explained differences in fertility, we expect to see no difference in recent sexual activity between HIV positive and HIV negative women. Since contraceptive use may differ between HIV positive and HIV negative women we also study exposure to recent sex without contraceptive use. We make the following assumptions:

- The relationship between the binary measure of sex in the last four weeks (yes/no) and an integer measure of coital frequency is the same for HIV positive and HIV negative women at the population level
- For an individual current contraceptive use is equivalent to contraceptive use in the last four weeks
- Contraceptive use efficacy is equal for HIV positive and HIV negative women.

We test our assumptions about recent sex as a proxy for coital frequency by repeating the analyses outlined below restricting it to married women, as marital status would likely affect the relationship between recent sex and coital frequency, so if marital patterns are different by HIV status, this relationship may also differ.

### Data

We analysed data from 46 Demographic and Health surveys (DHS) and AIDS indicator surveys (AIS) from 26 countries in sub-Saharan Africa that included both HIV testing data and questions about recent sexual intercourse and current contraceptive use (Table 1). Four surveys with HIV testing (Tanzania 2008 and 2012, Cote D’Ivoire 2005 and Uganda 2011) were excluded as they did not include questions on current contraceptive use.

**Table 1:** Summary of Demographic and Health surveys used.

Region	Survey	Year	n	HIV prevalence Women 15–49 (95% CI)*	Estimated^†^ female adults 15+ ART coverage^†^ (%)^†^(18)
**Southern Africa**					
	Lesotho	2004	3030	26.3 (24.5–28.2)	1 (1–1)
	Lesotho	2009	3778	26.7 (25.0–28.6)	27 (25–29)
	Lesotho	2014	3175	29.7 (27.7–31.8)	40 (37–43)
	Namibia	2013	4051	16.9 (15.4–18.4)	62 (50–70)
	Swaziland	2006–07	4424	31.1 (29.4–32.9)	10 (8–11)
	Zimbabwe	2005–06	6947	21.1 (19.7–22.6)	2 (2–3)
	Zimbabwe	2010–11	7313	17.7 (16.6–18.8)	31 (24–38)
	Zimbabwe	2015	8667	16.7 (15.6–17.8)	72 (57–84)
**East and Mid Africa**					
	Burundi	2010	4533	1.7 (1.4–2.1)	33 (26–40)
	Kenya	2003	3151	8.7 (7.6–10.0)	0 (0–0)
	Kenya	2008–09	3641	8 (6.8–9.3)	16 (15–18)
	Malawi	2004	2686	13.3 (12.0–14.8)	1 (1–2)
	Malawi	2010	7091	12.9 (11.8–14.1)	31 (29–33)
	Malawi	2015–16	7737	10.8 (9.9–11.7)	66 (63–71)
	Rwanda	2005	5641	3.6 (3.1–4.2)	9 (8–11)
	Rwanda	2010	6917	3.7 (3.3–4.2)	45 (39–51)
	Rwanda	2014–15	6752	3.6 (3.2–4.1)	67 (59–76)
	Zambia	2007	5502	16.1 (14.7–17.5)	20 (19–22)
	Zambia	2013–14	14719	15.1 (14.2–16.0)	53 (50–56)]
**West and Central Africa and Ethiopia**					
	Burkina	2003	4086	1.5 (1.2–2.0)	1 (1–1)
	Burkina	2010	8298	1.2 (0.9–1.5)	32 (25–40)
	Cameroon	2004	5128	6.6 (5.9–7.4)	2 (2–3)
	Cameroon	2011	7221	5.6 (5.0–6.3)	18 (15–20)
	Chad	2014–15	5656	1.8 (1.4–2.2)	50 (42–59)
	Cote Ivoire	2011–12	4509	4.6 (3.9–5.4)	25 (22–27)
	DRC	2007	4492	1.6 (1.2–2.2)	5 (4–6)
	DRC	2013–14	9264	1.6 (1.2–2.2)	24 (19–29)
	Ethiopia	2005	5736	1.9 (1.4–2.4)	2 (2–3)
	Ethiopia	2011	14695	1.9 (1.5–2.3)	41 (32–51)
	Gabon	2012	5459	5.8 (4.7–7.1)	32 (26–38)
	Gambia	2013	4089	2.1 (1.6–2.8)	24 (18–30)
	Ghana	2003	5097	2.3 (1.9–2.8)	0 (0–0)
	Guinea	2005	3742	1.9 (1.4–2.6)	2 (1–2)
	Guinea	2012	4622	2.1 (1.7–2.6)	28 (21–34)
	Liberia	2007	6382	1.8 (1.4–2.1)	3 (2–3)
	Liberia	2013	4397	2 (1.5–2.8)	19 (15–24)
	Mali	2006	4528	1.4 (1.0–2.0)	8 (6–10)
	Mali	2012–13	4806	1.3 (1.0–1.8)	32 (24–40)]
	Niger	2006	4406	0.6 (0.4–0.9)	3 (2–4)
	Niger	2012	5000	0.4 (0.2–0.5)	27 (20–32)
	Sao Tome	2009	2378	1.3 (0.8–2.0)	.
	Senegal	2005	4229	0.7 (0.4–1.0)	0 (0–0)
	Senegal	2010–11	5326	0.6 (0.4–0.8)	33 (25–40)
	Sierra Leone	2008	3448	1.7 (1.3–2.3)	4 (3–5)
	Sierra Leone	2013	7695	1.7 (1.3–2.0)	21 (13–29)
	Togo	2013–14	4737	3.1 (2.6–3.7)	37 (27–49)

* Estimated HIV prevalence, see Methods section

^†^ http://aidsinfo.unaids/, accessed 07 September 2017. Note for those surveys running over two years the earlier year is given

#### Outcome variables

*Exposure to sex*: We created a binary variable “had recent sexual intercourse” defined as reporting having had sexual intercourse in the last four weeks.

*Married*: Marital status was defined as a binary outcome: currently married (including cohabiting couples) and not currently married.

*Modern Contraceptive use*: Modern contraceptive use conformed to the DHS definition and included the pill, IUD, injections, diaphragm, condom, female sterilization, male sterilization, implants, female condom, Foam/Jelly and lactational amenorrhea. We restricted lactational amenorrhea to be included only if it was within six months of the birth.

*Exposure to pregnancy*: A binary outcome “exposure to pregnancy” was calculated as those who reported recent intercourse and reported not to be currently using any modern contraceptive. This definition assumes that current contraceptive use was constant in the 4 weeks prior to the survey.

*Condom use*: This binary outcome defined as women reporting currently using condoms among women who had reported recent sex and currently using a modern contraceptive.

*Fertility Rates*: This is measured using the retrospective birth histories and calculated as births per person year in the three years preceding the survey.

#### Explanatory variables

Other variables included women’s HIV status at the time of the survey, five-year age group at time of survey, calendar year, place of residence (urban/rural), geographic region, and national female ART coverage in the year of the survey drawn from UNAIDS estimates [14] and stratified into categories <20%, 20–49%, and >50%. Region was grouped into Southern (Zimbabwe, Lesotho, Swaziland and Namibia), East and Mid Africa (Tanzania, Kenya, Uganda, Rwanda, Burundi, Malawi and Zambia) and West and Central Africa with Ethiopia (Table 1). HIV epidemics in the East and Mid African countries occurred earlier than in Southern Africa. West and central Africa along with Ethiopia have lower prevalence and their HIV transmission is likely to be more concentrated in high risk groups.

Women who were pregnant at the time of the survey (9.0% across all surveys); those infected with HIV-2 (0.04%); and those whose HIV test was indeterminate (0.02%) were excluded from the analysis.

### Data analysis

For the outcome variables recent sex and recent exposure to pregnancy, we used log binomial regression to estimate the interaction between HIV status and five-year age group, place of residence, region and national ART coverage. Design-based variance estimates accounted for complex survey design. Each model was adjusted for country and survey year.

We further analysed the outcomes of modern contraceptive use and marital status using the same log binomial regression models to evaluate the extent to which differences in exposure to pregnancy between HIV positive and HIV negative women were mediated by differences in marital status or contraceptive use. Finally, we investigated differences in contraceptive type between HIV positive and HIV negative women and the potential implications of this for contraceptive efficacy. We assessed differences in type of modern contraceptives used between HIV positive and HIV negative women by analysing differences in condom use among women who reported having recent sex and currently using a modern contraceptive.

For fertility rate ratios we used exponential regression with the same covariates described above. Fertility data for the three years before the survey were modelled, with an additional categorical variable for each year before the survey interacted with the age groups below 25 years and above 25 years^3^. Estimated fertility rate ratios by HIV status pertain to estimated fertility rate for the year preceding the survey.

Relative exposure to pregnancy by HIV status were compared to the fertility rate ratios by HIV status in order to estimate how much of the reduced fertility in HIV positive women compared to HIV negative women at the population level could be attributed to less exposure to sex.

All analysis account for the two-stage cluster sampling survey design and use the HIV weights provided by the DHS. Surveys are reweighted so that each survey contributes equally towards the analysis. Analysis was conducted using Stata 15.1.

#### Decomposition of fertility differences

Due to the cross-sectional nature of the data we are unable to directly measure the contribution of recent sex to differences in fertility by HIV status as the outcome. The only possible measures of fertility relate to births in the years before the survey, so they come before the exposure, sex in the last four weeks. Instead, we have estimated relative fertility rates and relative exposure to pregnancy between HIV positive and HIV negative women within a given age, location, and time period. Comparing these risk ratios allows us to decompose the fertility differences by HIV status into differences in exposure to pregnancy and inferred differences in fecundity, as shown below.

The probability of having a live birth for an HIV negative women is:

$$F_{-ve}{=E}_{-ve}\times B_{-ve}$$

$$∴B_{-ve}=\frac{F_{-ve}}{E_{-ve}}$$ (1)

Where *E*_−*ve*_ is the probability of being sexually exposed to pregnancy and *B*_−*ve*_ the probability of becoming pregnant and having a live birth given exposure.

For an HIV positive women

$$F_{+ve}{=E}_{+ve}\times B_{+ve}$$

$$F_{+ve}{=E}_{+ve}\times B_{-ve}\times \beta$$ (2)

Where *β* is the additional risk factor of being HIV positive.

Rearranging (2) and substituting in (1)

$$\beta =\frac{F_{+ve}}{E_{+ve}\times B_{-ve}}=\frac{F_{+ve}\times E_{-ve}}{F_{-ve}\times E_{+ve}}$$

$$\approx \frac{Fertility\;rate\;ratio\;(+ve/-ve)}{Risk\;ratio\;of\;exposure\;to\;pregnancy\;(+ve/-ve)}$$

To obtain estimates of the relative difference in fecundity for HIV positive women, we use the risk ratios of being exposed to pregnancy analysed in this analysis along with the fertility rate ratios from Marston et al [3].

### Ethical approval

Ethical approval was obtained from the London School of Hygiene and Tropical Medicine ethics committee 2nd May 2017. DHS obtained the required local ethical approval and permission for each survey.

## Results

### Recent sex by HIV status

**Fig 2:**
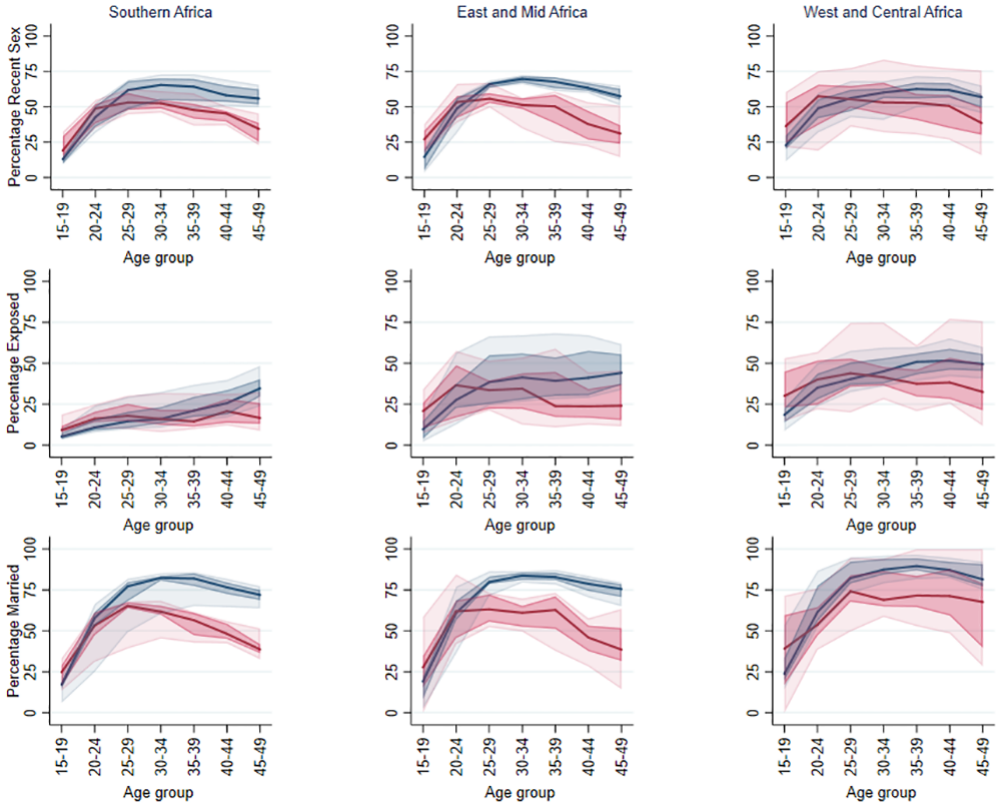
Cross-survey median percentages for recent sex, exposure to pregnancy (Exposed) and being married by HIV status (blue negative, red positive) by HIV status and region. Also shown is the interquartile range and the 10^th^ to 90^th^ percentile range.

In Eastern, Mid and Southern Africa the median percentage of women reporting recent sex across surveys was around 25% at age 15–19. This peaked in the age range 25–34 at around 70% in HIV negative women and 50% in HIV positive women and then declined after this age. For West and Central Africa the peak occurred at slightly older ages, in 30–39 year olds (Fig 2).

**Fig 3:**
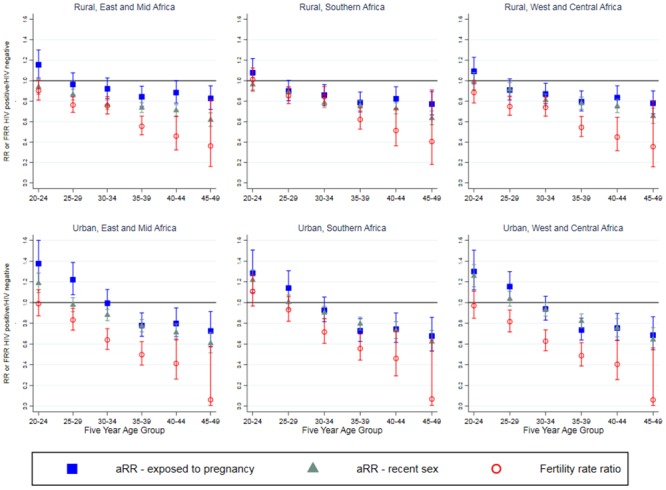
Adjusted risk ratio for recent sex and exposure to pregnancy, comparing HIV positive women to negative women (derived from models 2 and 5) with fertility rate ratio. Results shown for age groups 20–24 to 45–49.

Differences in recent sex between HIV positive and HIV negative women varied with age, residence and region, although the general patterns are similar. Among younger women, HIV positive women were more likely to have had recent sex compared to HIV negative women. This relationship switched around age 20–24 for rural women and 25–29 for urban women after which HIV positive women were less likely to report recent sex than HIV negative women (See Table 2, model 2, Fig 3 and S1 Appendix table B). In Southern and Western and Central Africa, the difference between HIV positive and HIV negative women was slightly smaller than in Eastern and Mid Africa (interaction term 1.04 95%CI 1.00–1.09 for Southern Africa and 1.05 95%CI 0.99–1.11 for Western and Central Africa) although this did not reach statistical significance for Western and Central Africa. This translates into small differences in each age group, for example, in the 30–34 year age group, East and Mid African HIV positive women in urban areas were 0.89 (95%CI 0.84–0.95) less likely to have had recent sex compared to 0.92 (95%CI 0.86–0.98) in Southern Africa. There was no evidence of variation of an interaction between HIV status and region by age. There was no evidence of any change in the relative probability of recent sex between HIV positive women to HIV negative women by ART coverage (Table 2, model 3).

**Table 2:** Adjusted risk ratios of recent sex, using Log Binomial model (see Fig 3 for derived risk ratios).

	Model 1*		Model 2*		Model 3*	
	FRR	95%CI	FRR	95%CI	FRR	95%CI
*HIV status*						
HIV negative	1		1		1	
HIV Positive	0.84	(0.81–0.87)	0.88	(0.83–0.92)	0.88	(0.82–0.94)
*Effects of HIV by age*						
15–19, HIV positive	1.73	(1.52–1.96)	1.72	(1.51–1.95)	1.55	(1.24–1.94)
20–24, HIV positive	1.25	(1.18–1.33)	1.25	(1.17–1.32)	1.31	(1.19–1.43)
25–29, HIV positive	1.11	(1.05–1.17)	1.10	(1.04–1.16)	1.10	(1.02–1.19)
30–34, HIV positive	1		1		1	
35–39, HIV positive	0.93	(0.88–0.99)	0.93	(0.88–0.99)	0.89	(0.82–0.98)
40–44, HIV positive	0.87	(0.81–0.93)	0.87	(0.81–0.93)	0.82	(0.73–0.92)
45–49, HIV positive	0.75	(0.68–0.82)	0.75	(0.68–0.82)	0.71	(0.61–0.83)
*Effects of HIV by Place of residence*						
rural, HIV positive			0.90	(0.86–0.93)	0.86	(0.79–0.93)
*Effects of Place of residence on age and HIV status interaction*						
rural, HIV positive,15–19					1.26	(0.96–1.66)
rural, HIV positive,20–24					0.97	(0.85–1.09)
rural, HIV positive,25–29					1.03	(0.93–1.15)
rural, HIV positive,30–34					1	
rural, HIV positive,35–39					1.11	(0.98–1.25)
rural, HIV positive,40–44					1.15	(0.99–1.32)
rural, HIV positive,45–49					1.12	(0.93–1.36)
*Effects of HIV by Region*						
Southern, HIV positive			1.04	(1.00–1.09)	1.04	(1.00–1.09)
East and Mid, HIV positive			1		1	
West and Central, HIV positive			1.05	(0.99–1.11)	1.05	(0.99–1.11)
*Effects of HIV by ART Coverage*						
<20%, HIV positive					1	
20–49%, HIV positive					1.02	(0.97–1.06)
>50%, HIV positive					1.00	(0.95–1.05)
*Age Group*						
15–19	0.29	(0.28–0.30)	0.29	(0.28–0.30)	0.26	(0.25–0.28)
20–24	0.75	(0.74–0.76)	0.75	(0.74–0.77)	0.68	(0.66–0.71)
25–29	0.95	(0.93–0.96)	0.95	(0.93–0.96)	0.91	(0.88–0.93)
30–34	1		1		1	
35–39	1.01	(0.99–1.03)	1.01	(0.99–1.02)	1.00	(0.97–1.03)
40–44	0.98	(0.97–1.00)	0.98	(0.97–1.00)	0.95	(0.91–0.98)
45–49	0.90	(0.88–0.91)	0.89	(0.88–0.91)	0.87	(0.83–0.91)
*Place of residence*						
urban					1	
rural			1.06	(1.05–1.08)	1.01	(0.98–1.03)
*Effects of age by Place of residence*						
rural, 15–19					1.15	(1.08–1.22)
rural, 20–24					1.15	(1.10–1.19)
rural, 25–29					1.06	(1.03–1.10)
rural, 30–34					1	
rural, 35–39					1.01	(0.97–1.05)
rural, 40–44					1.05	(1.01–1.09)
rural, 45–49					1.04	(0.99–1.09)
*ART Coverage*						
<20%					1	
20–49%					0.91	(0.86–0.96)
>50%					0.93	(0.84–1.02)

*All models also adjusted for calendar year (categorical) and country

#### Differences in modern contraceptive use for those who have had recent sex

Reported use of current modern contraceptive use among women who reported having had recent sex was substantially higher in Southern Africa compared to the other regions, with lowest reported use in Western and Central Africa. Cross survey median percentages for ages 20–29 were 71%,43% and 21% for Southern, East and Mid and West and central Africa respectively (S1 Appendix, Fig B).

Among women who reported recent sex, overall HIV positive women were less likely to report current use of modern contraceptives (S1 Appendix Table C, Fig C). However, these differences varied by age, region and place of residence. In urban areas for Southern and East and Mid Africa HIV positive women under 35 years old reported lower modern contraceptive use than HIV negative women (aRR ranging from 0.68, 95%CI 0.61–0.77 to 0.94, 95%CI 0.89–0.99). This pattern was similar in East and Mid Africa for older women but in Southern African women over 35 years old there was little or no difference in contraceptive use. In West and Mid Africa there was no evidence of a difference between current modern contraceptive use comparing HIV positive women to HIV negative women. In rural areas the pattern is different. Among younger women there was very little evidence of any difference between HIV positive and negative women’s current contraceptive use. For East and Mid Africa the risk ratios were below one for all ages indicating lower use of modern contraceptives among HIV positive women but this only reaches statistical significance (p<0.05) at ages 25 to 34 years. Among rural women older than 35 in southern Africa, HIV positive women are more likely to be currently using modern contraceptives than HIV negative women (for example 35–39 year old aRR is 1.08 (95%CI 1.01–1.16) (see S1 Appendix tables C-D, fig C).

#### Differences in condom use amongst modern contraceptive users

Of the women reporting having had recent sex and currently using modern contraceptives, HIV positive women were more likely to be using condoms than HIV negative women at all ages. The magnitude of this difference varied by age, region and rural and urban residency (S1 Appendix Fig D).

#### Recent exposure to pregnancy

Relative differences in recent exposure to pregnancy between HIV positive and HIV negative women were different from the relative patterns in recent sex, due to HIV positive women in many exposure groups having lower use of modern contraceptives than their HIV negative counterparts. Fig 2 shows the cross-survey median percentages of exposure to pregnancy by HIV status and region.

Rural HIV positive women aged 15–24 had a higher risk of recent exposure to pregnancy compared to HIV negative women (Fig 3). This switched around age 25–34 where HIV positive women are less exposed to pregnancy than the HIV negative women with a general trend of an increase in this gap with age. There was borderline evidence for regional variation with a slightly increased difference in recent exposure to pregnancy between HIV positive and HIV negative women in Southern and West and Central Africa compared to East and Mid Africa, with interaction terms 0.95 (95%CI 0.87–1.04) and 0.93 (95%CI 0.85–1.01), respectively. There was no evidence of a variation of region and HIV by age, though statistical power was limited to detect such an interaction. Young urban women also had higher exposure to pregnancy at young ages and lower exposure at older ages, but the crossover occurred at an older age group of around age 30–39, with some regional variation. There was no evidence of change in the relationship of HIV positive women to HIV negative women’s exposure to pregnancy by ART coverage (Table 3, model 6).

**Table 3:** Risk ratios of exposure to pregnancy, using log binomial model (see Fig 3 for derived risk ratios).

	Model 4*		Model 5*		Model 6*	
	FRR	95%CI	FRR	95%CI	FRR	95%CI
*HIV status*						
HIV negative	1		1		1	
HIV Positive	0.90	(0.83–0.97)	1.01	(0.89–1.14)	1.00	(0.88–1.15)
*Effects of HIV by age*						
15–19, HIV positive	1.85	(1.52–2.24)	1.78	(1.24–2.56)	1.79	(1.25–2.58)
20–24, HIV positive	1.29	(1.15–1.44)	1.38	(1.16–1.65)	1.38	(1.15–1.64)
25–29, HIV positive	1.12	(1.01–1.24)	1.23	(1.05–1.44)	1.23	(1.05–1.44)
30–34, HIV positive	1		1		1	
35–39, HIV positive	0.86	(0.76–0.96)	0.79	(0.66–0.94)	0.79	(0.66–0.94)
40–44, HIV positive	0.90	(0.80–1.01)	0.81	(0.66–0.98)	0.82	(0.67–0.99)
45–49, HIV positive	0.84	(0.73–0.96)	0.73	(0.57–0.94)	0.74	(0.58–0.94)
*Effects of HIV by Place of residence*						
rural, HIV positive			0.93	(0.80–1.08)	0.92	(0.79–1.07)
*Effects of Place of residence on age and HIV status interaction*						
rural, HIV positive,15–19			1.06	(0.69–1.63)	1.07	(0.70–1.64)
rural, HIV positive,20–24			0.90	(0.72–1.13)	0.91	(0.73–1.14)
rural, HIV positive,25–29			0.85	(0.69–1.05)	0.84	(0.68–1.04)
rural, HIV positive,30–34			1		1	
rural, HIV positive,35–39			1.16	(0.92–1.46)	1.16	(0.92–1.46)
rural, HIV positive,40–44			1.19	(0.93–1.53)	1.18	(0.92–1.52)
rural, HIV positive,45–49			1.23	(0.92–1.65)	1.22	(0.91–1.64)
*Effects of HIV by Region*						
Southern, HIV positive			0.95	(0.87–1.04)	0.95	(0.87–1.05)
East and Mid, HIV positive			1		1	
West and Central, HIV positive			0.93	(0.85–1.01)	0.93	(0.85–1.02)
*Effects of HIV by ART Coverage*						
<20%, HIV positive					1	
20–49%, HIV positive					1.02	(0.94–1.10)
>50%, HIV positive					1.02	(0.92–1.13)
*Age Group*						
15–19	0.33	(0.32–0.34)	0.28	(0.26–0.31)	0.28	(0.26–0.30)
20–24	0.75	(0.73–0.77)	0.66	(0.62–0.70)	0.66	(0.62–0.70)
25–29	0.94	(0.91–0.96)	0.87	(0.83–0.92)	0.87	(0.83–0.92)
30–34	1					
35–39	1.07	(1.04–1.10)	1.09	(1.03–1.15)	1.09	(1.03–1.15)
40–44	1.12	(1.09–1.15)	1.14	(1.07–1.20)	1.12	(1.06–1.19)
45–49	1.12	(1.09–1.15)	1.12	(1.05–1.19)	1.13	(1.06–1.20)
*Place of residence*						
urban			1		1	
rural			1.21	(1.16–1.27)	1.23	(1.18–1.29)
*Effects of age by Place of residence*						
rural, 15–19			1.30	(1.19–1.42)	1.29	(1.18–1.41)
rural, 20–24			1.24	(1.17–1.32)	1.24	(1.16–1.32)
rural, 25–29			1.12	(1.05–1.19)	1.12	(1.05–1.19)
rural, 30–34			1		1	
rural, 35–39			0.98	(0.92–1.04)	0.97	(0.91–1.03)
rural, 40–44			0.97	(0.91–1.04)	0.98	(0.92–1.04)
rural, 45–49			0.98	(0.92–1.05)	0.97	(0.91–1.04)
*ART Coverage*						
<20%					1	
20–49%					0.87	(0.78–0.98)
>50%					0.78	(0.67–0.91)

*All models also adjusted for calendar year and country

#### Estimating fertility rate ratios if exposure was the same for recent sex and exposure to pregnancy

**Fig 4:**
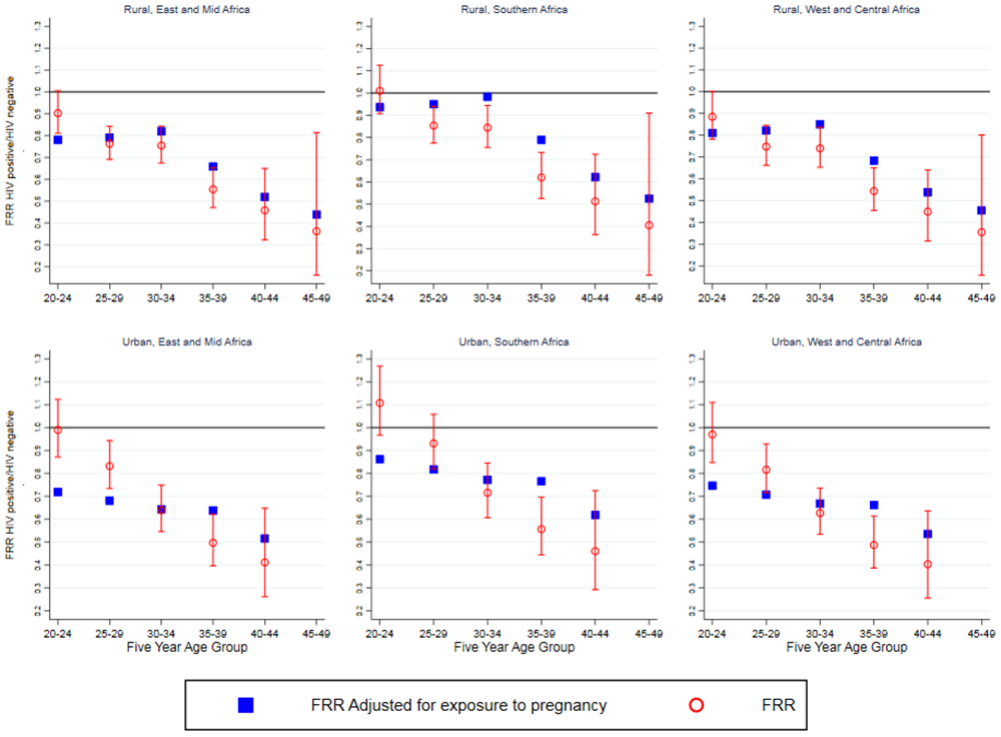
Adjusted fertility rate ratios (FRR) with adjusted fertility rate ratios adjusted for exposure to pregnancy (FRR/RR).

Fig 4 shows the inferred relative fecundity of HIV positive women compared to HIV negative, when adjusted for differences in exposure to pregnancy. The estimated fecundity for HIV positive women under 35 was between 0.6 and 0.9 compared to that of HIV negative women. This varied by place of residence and region with greater reductions in fertility in urban areas compared to rural areas. The estimated fecundity reduction was less in Southern Africa compared to the two other regions. After the age of 35 the estimated fecundity reduction intensified gradually as age increases.

#### Marriage

Fig 2 shows the median percentage married across surveys by age, HIV status and region. These showed a slightly higher or similar median percentage of women married among HIV positive women up to the ages of 20–24. After this age HIV negative women were more likely to be married with the gap increasing with age.

Rural HIV positive women aged 20–49 are less likely to report being currently married compared to HIV negative women with this difference widening as age increases (S1 Appendix tables G-H, fig G). For example in Southern Africa, the relative probability of being married for HIV positive women was 0.85 (95% CI 0.80–0.90) at 20–24 years old to 0.61 (95% CI 0.56–0.66) at age 45–49. Young urban HIV positive women below the age of 30 have a higher or similar risk of being married compared to their HIV negative counterparts but after 30 their probability of marriage becomes lower than that of HIV negative women, with the relative difference increasing with age. There is some regional variation with Western and Central Africa having a slightly reduced difference. There is some evidence that the gap between positive and negative women has narrowed with increased ART coverage.

#### Married women differences between recent sex and exposure to pregnancy

When restricted to married women only, differences in recent sexual activity were much smaller between HIV positive women and HIV negative women than when considering all women (S1 Appendix tables I-L, figs H-K). For rural married women there was no evidence of a difference by HIV status for ages 20–34 and a slightly lower risk of recent sex for HIV positive married women aged 35–44. However, for rural HIV positive women there was a higher risk of exposure to a pregnancy for women of all ages apart from 35–39 year olds. For West Africa the pattern was the same but differences were not statistically significant. For urban married women aged 20–34 in Southern Africa there was evidence of an increased risk of recent sex compared to HIV negative married women, with no evidence of a difference at older ages 35–44. This pattern was the same in the other regions but did not reach statistical significance.

#### Estimating the fecundity reduction for married women

Estimates of the fecundity reduction estimated among married women only gave broadly similar results as for all women although implying slightly larger fecundity reduction for HIV positive rural women in Eastern and Mid and Southern Africa (S1 Appendix figs L-M). Detailed results are further reported in the appendix (S1 Appendix), including all models, stratified rate ratios and graphs.

## Discussion

This analysis has shown that patterns of sexual exposure between HIV positive and HIV negative women by age, region and place of residence strongly mirror the patterns of relative fertility rates between the two groups. We have also shown that after adjusting for differences in exposure to recent sexual activity, young HIV positive women may have somewhat lower rates of conception and fertility than HIV negative counterparts, similar to that observed among women in older age groups.

Many studies have found higher fertility rates in young HIV positive women 15–19 compared to HIV negative women. This is due to selection effects where women who begin sexual activity are exposed to both the risk of pregnancy and HIV [1–3, 15]. In settings where median age at first sex is higher these selection effects can also have some impact in the 20–24 year age group [3]. At older ages, studies have found that HIV positive women experience lower fertility compared to HIV negative women, including due to widowhood and separation from a partner, or lower sexual activity due to illness [12]. This analysis confirms both these aspects of sexual activity. Rural HIV positive women have an increasingly lower probability of recent sex as age increases and urban HIV positive women have a higher risk of recent sex in 20–24 year olds followed by a lower risk after the age of 30.

A different pattern emerges when examining exposure to pregnancy, accounting for both sexual activity and modern contraceptive use. Modern contraceptive use is lower among HIV positive women, resulting in higher exposure to pregnancy for younger HIV positive women compared to HIV negative women up to the age of around 25 years old for rural areas and 30 for urban areas. For older women the exposure to pregnancy remains lower for HIV positive women than HIV negative women although their risk of recent exposure to pregnancy was slightly closer than their risk of recent sex. These differences in recent exposure to pregnancy appear to disguise the direct effects of HIV on fertility. With the assumptions that the relationship between recent sex and coital frequency is the same at population level for HIV positive and HIV negative women and that the effectiveness of contraceptive use is the same across the two groups, we estimate that the impact of HIV on fecundity is much higher in younger women than might be gauged from HIV-associated subfertility measured in general populations, without taking into account sexual behaviour [1–3] and slightly lower in older women.

Taken together, this suggests that the direct effects of HIV on fertility are more similar across ages, while the dramatic age gradient that has frequently been observed is largely attributable to variation in relative sexual exposure by age. We estimate that in these younger women, if biological factors impacting fecundity were the only effect on fertility, the fertility risk ratio comparing HIV positive women to HIV negative women would be around 0.7 to 0.9. This appears to vary by region and urban and rural residency, with a greater estimated biological impact on fecundity in urban areas and a lower estimated biological impact in Southern Africa.

A possible explanation for a biological impact of HIV on fecundity in women at the earlier stages of HIV infection may be co-infection or past infection with other sexually transmitted infections (STI). The role of STIs in HIV subfertility has been discussed previously [16–18]. HIV positive women are more likely to be infected with another STI than HIV negative women as STI have similar risk factors to HIV. STI such as Chlamydia and Gonorrhoea cause Pelvic inflammatory disease that can impair fertility soon after infection and if left untreated can then go on to cause permanent infertility due to tubal damage [19]. Other STI such as syphilis are linked to adverse fetal outcomes: miscarriage and still birth [20]. These STIs in women are generally asymptomatic [21] and therefore may go untreated, but also lack of access to health facilities or social stigma may contribute to lack of treatment. Variation in biological subfertility by place of residence and region may be explained by the prevalence and type of STIs and access to treatment.

These results also shed light on the persistent fertility differences between HIV positive and HIV negative women in the era of ART [3, 9, 22]. Although it is possible that high coverage has not been sustained for long enough to observe a dramatic recovery in fertility yet, it is also possible that although ART improves the health of HIV positive women, it may not immediately change their prospects for sexual activity and exposure to pregnancy, or it may not ameliorate the long-term impacts of previous STIs on impaired fertility. There was no evidence of an effect of ART coverage or timing of ART roll-out in this analysis indicating that the relative difference in recent exposure to pregnancy between HIV positive and HIV negative women has not changed over time. There are few DHS studies in countries that have achieved a high ART roll out for a sustained amount of time, therefore it is possible that it is too soon to detect behavioural changes due to the presence of ART.

As this analysis uses cross sectional data we are unable to model sexual behaviour as an explanatory factor for individual-level fertility outcomes. Our measure of fertility comes from birth histories in the year before the survey, the explanatory variable recent sex relates to the four weeks prior to the survey, and current contraceptive use is measured at the time of the survey. Therefore our estimates of the biological effect of HIV on fecundity are indirect, depending on ratio comparisons of fertility rates and rates of exposure to unprotected sex.

For this analysis we assume that the relationship between recent sex and coital frequency is the same by HIV status of the women. However apart from 15–19 year olds, HIV positive women were generally less likely to be married than HIV negative women, therefore we might find that the relationship between recent sex and coital frequency may be different if the latter were to be measured directly. To reduce the possibility of this biasing our analysis, we also restricted the analysis to married women only, which yielded broadly similar results for the estimated effect of HIV on fertility. However coital frequency may also be determined by a variety of other factors such as partner mobility, polygamy, duration of marriage and desire for children which we have not yet investigated.

We used reported current modern contraceptive use as a proxy for the contraceptive behaviour during the four weeks prior to the survey. Although this may be a reasonable assumption it is possible that consistency and contraceptive efficacy varies by type of contraceptives. Of the women who had reported recent sex and currently using modern contraceptives, HIV positive women were more likely to be using condoms than HIV negative women. Condom are both less effective than other modern contraceptives as they are more likely to be incorrectly used and used differently with different partners so may be less likely to have been used consistently over the previous four weeks. With condom use more prevalent in HIV positive women it is possible that they have a higher exposure to pregnancy risk relative to HIV negative women, even if the exposure to pregnancy variable used in this analysis is similar. This may have more of an effect in areas where prevalence of condom use is higher. Additionally reporting of contraceptive use can be unreliable [23], which could effect the results if the unreliability of the reporting differed between HIV positive and HIV negative women.

We have shown that differences in sexual activity between HIV positive and HIV negative women by age largely explain the steep gradient in fertility rate ratios by age that has been previously described, as well as regional and urban/rural differences in relative fertility. Moreover, recent sexual activity and exposure to pregnancy for HIV positive women has not increased significantly since ART was scaled-up. This may go some way explain why we have not observed the rapid increases in fertility of HIV positive women that would be predicted by mathematical models which assume women on ART will have the same fertility as HIV negative women of the same age. We also hypothesize that long-term fertility impairment due to other STIs or lasting immunological effects of HIV may contribute to the continuing lower average fertility of HIV positive women. These dynamics could continue to evolve as both women and men initiate ART earlier, widowhood and marriage dissolution decrease, and norms around sexual behaviour and HIV continue to change.

## Supporting information

S1 Appendix (PDF)
