# Supplementary material for: Relative patterns of sexual activity and fertility among HIV positive and negative women—Evidence from 46 DHS
Source: PLoS One. 2018 Oct 17;13(10):e0204584. doi: 10.1371/journal.pone.0204584 (PMC6192566; doi:10.1371/journal.pone.0204584)

S1 Appendix - Relative patterns of sexual activity and fertility among HIV positive and negative women

Milly Marston [1*],Basia Zaba[1], Jeffrey W Eaton[2].

1 London School of Hygiene and Tropical Medicine, London, UK

2 Department of Infectious Disease Epidemiology, Imperial College, London, UK

*Corresponding Author: milly.marston@lshtm.ac.uk

Tables

Table A: Risk ratios of recent sex, using log binomial model 3
Table B: Stratum specific risk ratios from Model 2, recent sex 4
Table C: Risk of using a current modern use of contraceptives for those who have had recent sex, comparing HIV positive to HIV negative women..7
Table D: Stratified rate ratios for the risk of using a current modern use of contraceptives for those who have had recent sex, comparing HIV positive to HIV negative women 9
Table E: Risk ratios of recent exposure to a live birth, using log binomial model...12
Table F: Stratum specific risk ratios from Model 1, exposure to live birth 13
Table G: Risk ratios of being married using Log Binomial model 16
Table H: Stratum specific risk ratios of being married comparing HIV positive women to HIV negative women. 17
Table I: Risk ratios of recent sex among **married women**, using log binomial model.....................................19
Table J: Risk ratios of exposure to a live birth, among **married women,**, using log binomial model...........................20
Table K: Stratum specific risk ratios, recent sex, **married women**, exclude pregnancy 21
Table L: Stratum specific risk ratios, Exposed, **married women**, exclude pregnancy 21

Figures

Figure A: Risk ratio of recent sex, comparing HIV positive women to HIV negative women...5
Figure B: Cross-survey median percentages for reported current use of a modern contraceptive amongst those reporting recent sex for all women. Also shown is the interquartile range and the 10th to 90th percentile range. 6
Figure C: Risk ratio of currently using modern contraceptives for women reporting recent sex, comparing HIV positive women to HIV negative women 10
Figure D: Risk ratio of using a condom for women who report having recent sex and currently using a modern contraceptive (HIV+ve/HIV-ve)...11
Figure E: Risk ratio of exposure to pregnancy, comparing HIV positive women to HIV negative women 14
Figure F: Adjusted Risk ratio for recent sex and exposure to pregnancy, comparing HIV positive women to negative women with Fertility rate ratio 15
Figure G: Adjusted Risk ratio for being married, comparing HIV positive women to negative women. 18
Figure H: Adjusted Risk ratio for recent sex among **married women**, comparing HIV positive women to HIV negative women. 22
Figure I: Risk ratio of currently using modern contraceptives among **married women** reporting recent sex, comparing HIV positive women to HIV negative women..23
Figure J: Adjusted Risk ratio for exposure to pregnancy among **married women**, comparing HIV positive women to negative women. 24
Figure K: Adjusted Risk ratio for recent sex and exposure to a live birth, comparing HIV positive women to negative women with Fertility rate ratio for **married women**...25
Figure L: Adjusted Fertility rate ratio (FRR/RR) for **married women**...26
Figure M: Comparing fertility rate ratios adjusted for exposure to pregnancy for all women to married women.......27

RECENT SEX BY HIV STATUS

Table A: Risk ratios of recent sex, using log binomial model

	Model 1		Model 2		Model 3	
	FRR	95 %CI	FRR	95 %CI	FRR	95 %CI
HIV status						
HIV negative	1		1		1	
HIV Positive	0.84	(0.81-0.87)	0.88	(0.83-0.92)	0.88	(0.82-0.94)
Effects of HIV by age						
15-19, HIV positive	1.73	(1.52-1.96)	1.72	(1.51-1.95)	1.55	(1.24-1.94)
20-24, HIV positive	1.25	(1.18-1.33)	1.25	(1.17-1.32)	1.31	(1.19-1.43)
25-29, HIV positive	1.11	(1.05-1.17)	1.10	(1.04-1.16)	1.10	(1.02-1.19)
30-34, HIV positive	1		1		1	
35-39, HIV positive	0.93	(0.88-0.99)	0.93	(0.88-0.99)	0.89	(0.82-0.98)
40-44, HIV positive	0.87	(0.81-0.93)	0.87	(0.81-0.93)	0.82	(0.73-0.92)
45-49, HIV positive	0.75	(0.68-0.82)	0.75	(0.68-0.82)	0.71	(0.61-0.83)
Effects of HIV by Place of residence						
rural, HIV positive			0.90	(0.86-0.93)	0.86	(0.79-0.93)
Effects of Place of residence on age and HIV status interaction						
rural, HIV positive,15-19					1.26	(0.96-1.66)
rural, HIV positive,20-24					0.97	(0.85-1.09)
rural, HIV positive,25-29					1.03	(0.93-1.15)
rural, HIV positive,30-34					1	
rural, HIV positive,35-39					1.11	(0.98-1.25)
rural, HIV positive,40-44					1.15	(0.99-1.32)
rural, HIV positive,45-49					1.12	(0.93-1.36)
Effects of HIV by Region						
Southern, HIV positive			1.04	(1.00-1.09)	1.04	(1.00-1.09)
Eastern, HIV positive			1		1	
Western, HIV positive			1.05	(0.99-1.11)	1.05	(0.99-1.11)
Effects of HIV by ART Coverage						
<20%, HIV positive					1	
20-49%, HIV positive					1.02	(0.97-1.06)
>50%, HIV positive					1.00	(0.95-1.05)
Age Group						
15-19	0.29	(0.28-0.30)	0.29	(0.28-0.30)	0.26	(0.25-0.28)
20-24	0.75	(0.74-0.76)	0.75	(0.74-0.77)	0.68	(0.66-0.71)
25-29	0.95	(0.93-0.96)	0.95	(0.93-0.96)	0.91	(0.88-0.93)
30-34	1		1		1	
35-39	1.01	(0.99-1.03)	1.01	(0.99-1.02)	1.00	(0.97-1.03)
40-44	0.98	(0.97-1.00)	0.98	(0.97-1.00)	0.95	(0.91-0.98)
45-49	0.90	(0.88-0.91)	0.89	(0.88-0.91)	0.87	(0.83-0.91)
Place of residence						
urban					1	
rural			1.06	(1.05-1.08)	1.01	(0.98-1.03)
Effects of age by Place of residence						
rural, 15-19					1.15	(1.08-1.22)
rural, 20-24					1.15	(1.10-1.19)
rural, 25-29					1.06	(1.03-1.10)
rural, 30-34					1	
rural, 35-39					1.01	(0.97-1.05)
rural, 40-44					1.05	(1.01-1.09)
rural, 45-49					1.04	(0.99-1.09)
ART Coverage						
<20%					1	
20-49%					0.91	(0.86-0.96)
>50%					0.93	(0.84-1.02)

Table B: Stratum specific risk ratios from Model 2, recent sex

		Southern Africa	East and Mid	West and central
Urban				
	15-19	1.42 (1.13-1.78)	1.37 (1.09-1.72)	1.44 (1.15-1.80)
	20-24	1.24 (1.14-1.34)	1.20 (1.10-1.29)	1.25 (1.15-1.36)
	25-29	1.02 (0.95-1.09)	0.99 (0.92-1.06)	1.03 (0.97-1.11)
	30-34	0.92 (0.86-0.98)	0.89 (0.84-0.95)	0.93 (0.87-1.00)
	35-39	0.81 (0.75-0.88)	0.79 (0.73-0.85)	0.82 (0.76-0.89)
	40-44	0.75 (0.67-0.83)	0.72 (0.65-0.80)	0.75 (0.67-0.85)
	45-49	0.64 (0.54-0.75)	0.61 (0.52-0.72)	0.64 (0.54-0.76)
Rural				
	15-19	1.23 (0.97-1.57)	1.19 (0.93-1.52)	1.25 (0.98-1.59)
	20-24	1.07 (0.96-1.20)	1.04 (0.93-1.16)	1.09 (0.96-1.22)
	25-29	0.89 (0.80-0.98)	0.86 (0.77-0.95)	0.90 (0.81-1.00)
	30-34	0.80 (0.75-0.85)	0.77 (0.73-0.82)	0.81 (0.75-0.87)
	35-39	0.71 (0.63-0.79)	0.68 (0.61-0.76)	0.71 (0.64-0.80)
	40-44	0.65 (0.57-0.74)	0.63 (0.55-0.72)	0.65 (0.57-0.75)
	45-49	0.55 (0.46-0.66)	0.53 (0.45-0.64)	0.56 (0.46-0.67)

Figure A: Risk ratio of recent sex, comparing HIV positive women to HIV negative women

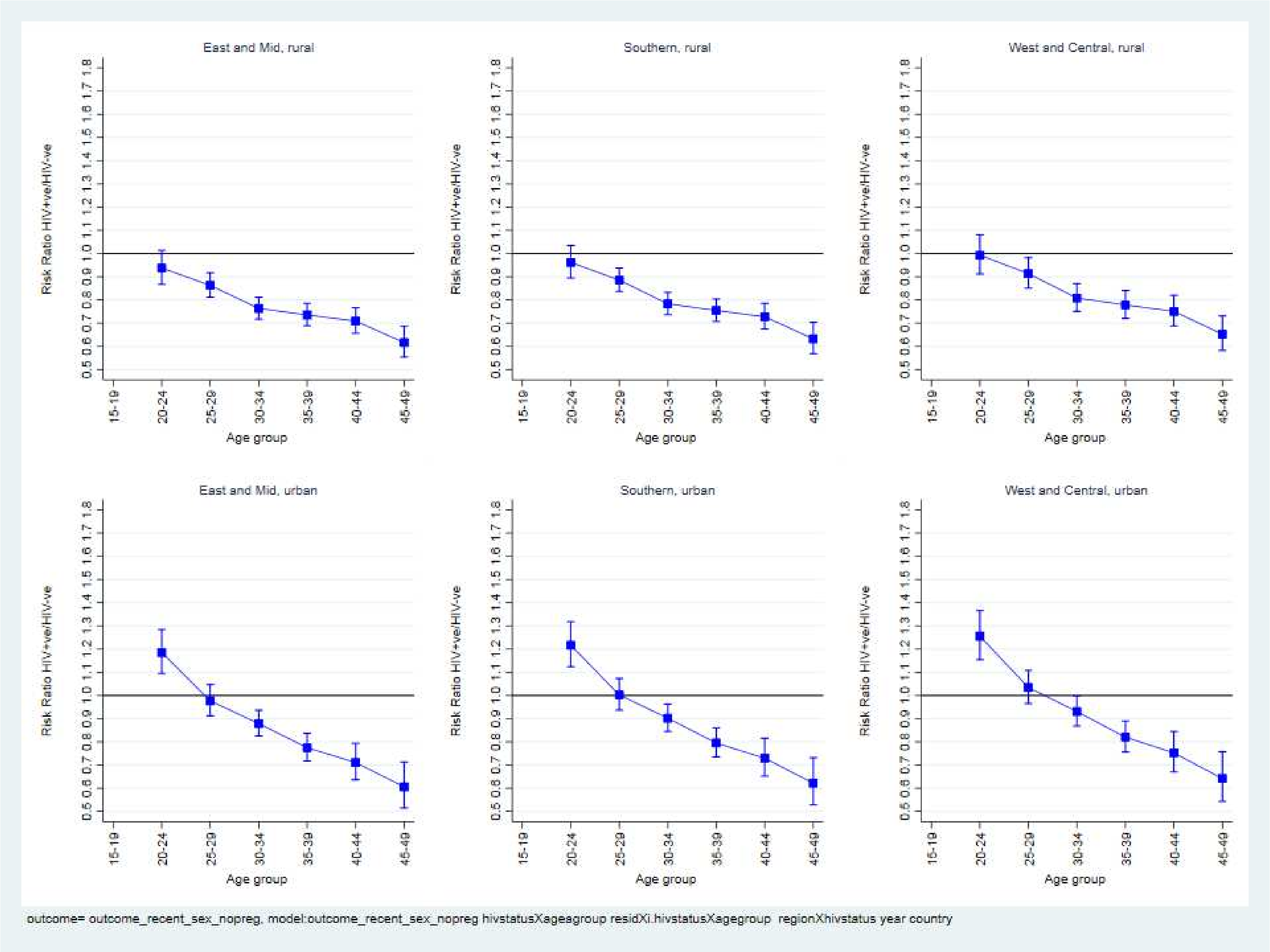

DIFFERENCES IN MODERN CONTRACEPTIVE USE FOR THOSE WHO HAVE HAD RECENT SEX

Figure B: Cross-survey median percentages for reported current use of a modern contraceptive amongst those reporting recent sex for all women. Also shown is the interquartile range and the 10th to 90th percentile range.

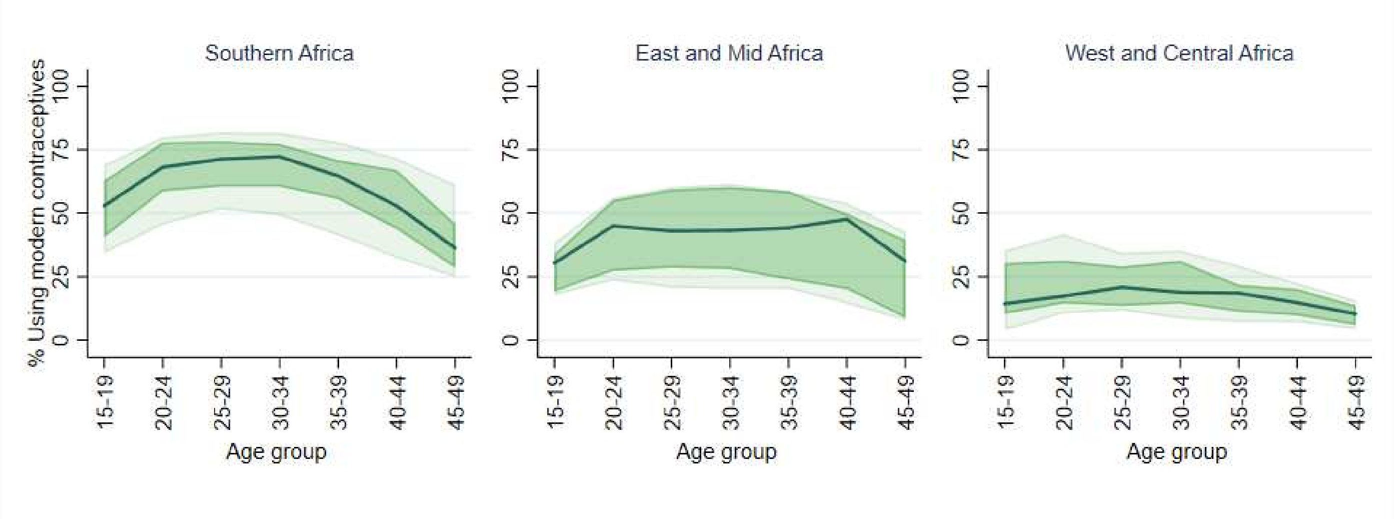

Table C: Risk of using a current modern use of contraceptives for those who have had recent sex, comparing HIV positive to HIV negative women

Current Modern use for recent sex	Model 1		Model 2		Model 3		Model 4	
	RR	95 %CI	RR	95 %CI	RR	95 %CI	RR	95 %CI
HIV status								
HIV negative	1		1		1		1	
HIV Positive	0.93(0.88-0.97)		0.68(0.61-0.77)		0.71(0.63-0.80)		0.69(0.60-0.78)	
Effects of HIV by age								
15-19, HIV positive	0.95(0.80-1.13)		1.23(0.88-1.73)		1.07(0.72-1.59)		1.06(0.72-1.57)	
20-24, HIV positive	0.95(0.88-1.03)		1.21(1.02-1.44)		1.23(1.02-1.49)		1.23(1.02-1.49)	
25-29, HIV positive	0.97(0.91-1.04)		1.12(0.95-1.32)		1.02(0.86-1.22)		1.03(0.86-1.23)	
30-34, HIV positive	1		1		1		1	
35-39, HIV positive	1.09(1.02-1.18)		1.22(1.03-1.44)		1.17(0.98-1.41)		1.16(0.97-1.40)	
40-44, HIV positive	1.05(0.95-1.16)		1.17(0.97-1.42)		1.11(0.88-1.40)		1.11(0.89-1.39)	
45-49, HIV positive	1.23(1.06-1.42)		1.15(0.87-1.53)		0.99(0.70-1.38)		0.98(0.70-1.37)	
Effects of HIV by Place of residence								
rural, HIV positive			1.22(1.15-1.29)		1.13(1.02-1.25)		1.13(1.02-1.26)	
Effects of Place of residence on age and HIV status interaction								
rural, HIV positive,15-19					1.22(0.85-1.74)		1.22(0.86-1.74)	
rural, HIV positive,20-24					0.97(0.83-1.15)		0.97(0.83-1.14)	
rural, HIV positive,25-29					1.19(1.04-1.37)		1.19(1.04-1.36)	
rural, HIV positive,30-34					1		1	
rural, HIV positive,35-39					1.06(0.92-1.23)		1.07(0.93-1.23)	
rural, HIV positive,40-44					1.09(0.89-1.33)		1.08(0.89-1.32)	
rural, HIV positive,45-49					1.24(0.91-1.68)		1.23(0.91-1.66)	
Effects of HIV by Region								
Southern, HIV positive			1.38(1.21-1.57)		1.35(1.19-1.53)		1.36(1.20-1.54)	
Western, HIV positive			1.36(1.00-1.83)		1.28(0.94-1.75)		1.33(0.97-1.81)	
Effects of Region on age and HIV status interaction								
Southern Africa, Positive, 15-19			0.72(0.48-1.08)		0.79(0.53-1.19)		0.80(0.54-1.20)	
Southern Africa, Positive, 20-24			0.75(0.61-0.91)		0.75(0.62-0.92)		0.76(0.63-0.92)	
Southern Africa, Positive, 25-29			0.83(0.70-1.00)		0.85(0.71-1.02)		0.85(0.71-1.01)	
Southern Africa, Positive, 30-34			1		1		1	
Southern Africa, Positive, 35-39			0.86(0.71-1.03)		0.88(0.73-1.06)		0.88(0.73-1.06)	
Southern Africa, Positive, 40-44			0.81(0.64-1.02)		0.83(0.66-1.04)		0.84(0.67-1.05)	
Southern Africa, Positive, 45-49			1.02(0.73-1.43)		1.08(0.78-1.51)		1.11(0.79-1.54)	
West and Central Africa, Positive, 15-19			1.05(0.55-2.02)		1.11(0.57-2.17)		1.12(0.58-2.18)	
West and Central Africa, Positive, 20-24			0.88(0.59-1.32)		0.96(0.64-1.44)		0.96(0.64-1.44)	
West and Central Africa, Positive, 25-29			0.90(0.61-1.33)		1.00(0.68-1.49)		1.00(0.67-1.47)	
West and Central Africa, Positive, 30-34			1		1		1	
West and Central Africa, Positive, 35-39			0.96(0.63-1.47)		1.03(0.67-1.59)		1.04(0.67-1.60)	
West and Central Africa, Positive, 40-44			1.10(0.67-1.81)		1.15(0.68-1.92)		1.15(0.69-1.92)	
West and Central Africa, Positive, 45-49			0.66(0.26-1.67)		0.51(0.19-1.36)		0.52(0.20-1.37)	
Effects of HIV by ART Coverage								
20-49%, HIV positive							1.00(0.93-1.06)	
>50%, HIV positive							1.07(1.01-1.14)	
Age Group								
15-19	0.81(0.77-0.84)		0.67(0.61-0.72)		0.81(0.73-0.89)		0.81(0.73-0.89)	
20-24	0.98(0.96-1.01)		0.91(0.87-0.95)		0.93(0.88-0.99)		0.93(0.88-0.99)	
25-29	1.01(0.99-1.03)		0.98(0.94-1.02)		0.99(0.94-1.04)		0.99(0.94-1.04)	
30-34	1		1		1		1	
35-39	0.91(0.88-0.94)		0.95(0.91-1.00)		0.99(0.93-1.04)		0.99(0.93-1.04)	
40-44	0.79(0.76-0.82)		0.86(0.81-0.90)		0.89(0.83-0.96)		0.89(0.83-0.96)	
45-49	0.53(0.50-0.56)		0.60(0.56-0.66)		0.76(0.69-0.85)		0.76(0.68-0.85)	
Place of residence								
urban			1		1		1	
rural			0.80(0.78-0.81)		0.83(0.80-0.86)		0.82(0.79-0.86)	
Effects of age by Place of residence								
rural, 15-19					0.75(0.68-0.82)		0.75(0.68-0.82)	
rural, 20-24					0.96(0.91-1.01)		0.96(0.91-1.01)	
rural, 25-29					0.98(0.93-1.03)		0.98(0.93-1.03)	
rural, 30-34					1		1	
rural, 35-39					0.95(0.90-1.01)		0.95(0.90-1.01)	
rural, 40-44					0.94(0.87-1.01)		0.94(0.87-1.01)	
rural, 45-49					0.73(0.66-0.81)		0.73(0.66-0.81)	

Effects of age by Region			
Southern Africa, 15-19	1.23(1.10-1.37)	1.13(1.01-1.26)	1.12(1.01-1.25)
Southern Africa, 20-24	1.09(1.03-1.15)	1.07(1.01-1.13)	1.07(1.01-1.13)
Southern Africa, 25-29	1.03(0.98-1.08)	1.02(0.97-1.08)	1.02(0.97-1.08)
Southern Africa, 30-34	1.00(1.00-1.00)	1.00(1.00-1.00)	1.00(1.00-1.00)
Southern Africa, 35-39	0.97(0.91-1.03)	0.95(0.89-1.01)	0.95(0.89-1.01)
Southern Africa, 40-44	0.98(0.91-1.06)	0.96(0.88-1.04)	0.96(0.88-1.03)
Southern Africa, 45-49	0.98(0.87-1.10)	0.92(0.82-1.03)	0.91(0.81-1.03)
West and Central Africa, 15-19	1.47(1.31-1.65)	1.44(1.29-1.61)	1.44(1.29-1.61)
West and Central Africa, 20-24	1.21(1.12-1.31)	1.20(1.11-1.30)	1.20(1.11-1.30)
West and Central Africa, 25-29	1.04(0.96-1.12)	1.02(0.95-1.10)	1.02(0.95-1.10)
West and Central Africa, 30-34	1.00(1.00-1.00)	1.00(1.00-1.00)	1.00(1.00-1.00)
West and Central Africa, 35-39	0.92(0.84-1.00)	0.90(0.83-0.98)	0.90(0.83-0.98)
West and Central Africa, 40-44	0.82(0.75-0.91)	0.82(0.74-0.91)	0.82(0.74-0.91)
West and Central Africa, 45-49	0.75(0.65-0.86)	0.71(0.62-0.82)	0.71(0.62-0.82)
ART Coverage			
<20%		1	1
20-49%		1.36 (1.17-1.58)	1.36 (1.17-1.59)
>50%		1.44 (1.08-1.91)	1.43 (1.08-1.90)

Table D: Stratified rate ratios for the risk of using a current modern use of contraceptives for those who have had recent sex, comparing HIV positive to HIV negative women

		Risk ratios by region		
		Southern Africa	East and Mid	West and central
Urban				
	15-19	0.84 (0.67-1.05)	0.84 (0.62-1.16)	1.20 (0.74-1.95)
	20-24	0.85 (0.78-0.93)	0.83 (0.72-0.95)	0.99 (0.77-1.27)
	25-29	0.88 (0.83-0.93)	0.77 (0.68-0.86)	0.94 (0.75-1.17)
	30-34	0.94 (0.89-0.99)	0.68 (0.61-0.77)	0.93 (0.70-1.22)
	35-39	0.99 (0.92-1.06)	0.83 (0.74-0.94)	1.08 (0.82-1.43)
	40-44	0.89 (0.79-1.01)	0.80 (0.68-0.94)	1.20 (0.83-1.73)
	45-49	1.11 (0.93-1.33)	0.79 (0.61-1.02)	0.71 (0.31-1.64)
Rural				
	15-19	0.92 (0.73-1.15)	0.93 (0.68-1.27)	1.32 (0.81-2.14)
	20-24	0.94 (0.86-1.02)	0.91 (0.80-1.04)	1.09 (0.85-1.39)
	25-29	0.96 (0.90-1.03)	0.84 (0.74-0.95)	1.03 (0.82-1.28)
	30-34	1.03 (0.98-1.10)	0.75 (0.67-0.85)	1.02 (0.77-1.34)
	35-39	1.08 (1.01-1.16)	0.91 (0.81-1.02)	1.19 (0.90-1.57)
	40-44	0.98 (0.87-1.10)	0.88 (0.75-1.03)	1.31 (0.91-1.90)
	45-49	1.22 (1.02-1.46)	0.86 (0.67-1.12)	0.78 (0.34-1.81)

Figure C: Risk ratio of currently using modern contraceptives for women reporting recent sex, comparing HIV positive women to HIV negative women

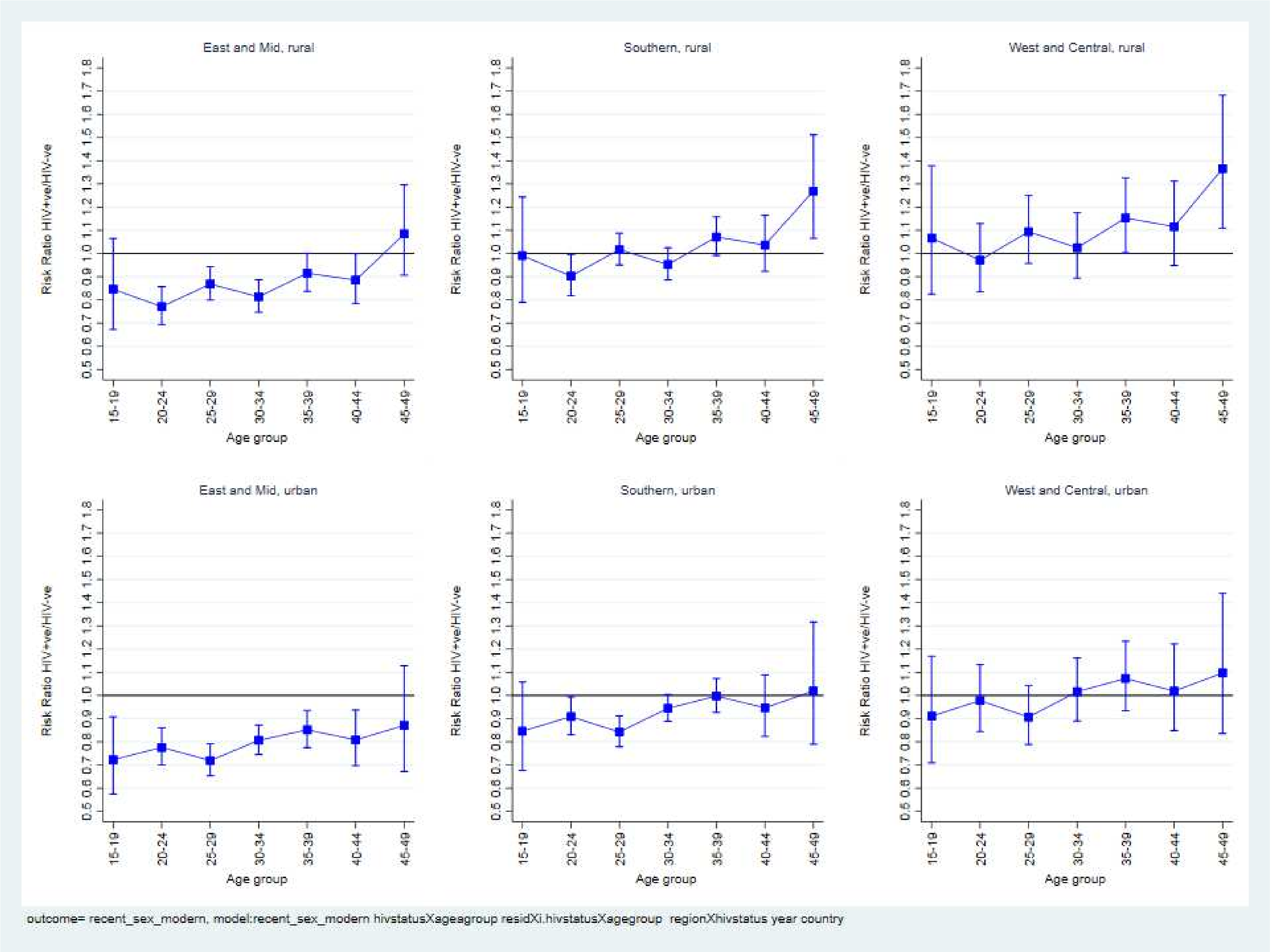

Figure D: Risk ratio of using a condom for women who report having recent sex and currently using a modern contraceptive (HIV+ve/HIV-ve)

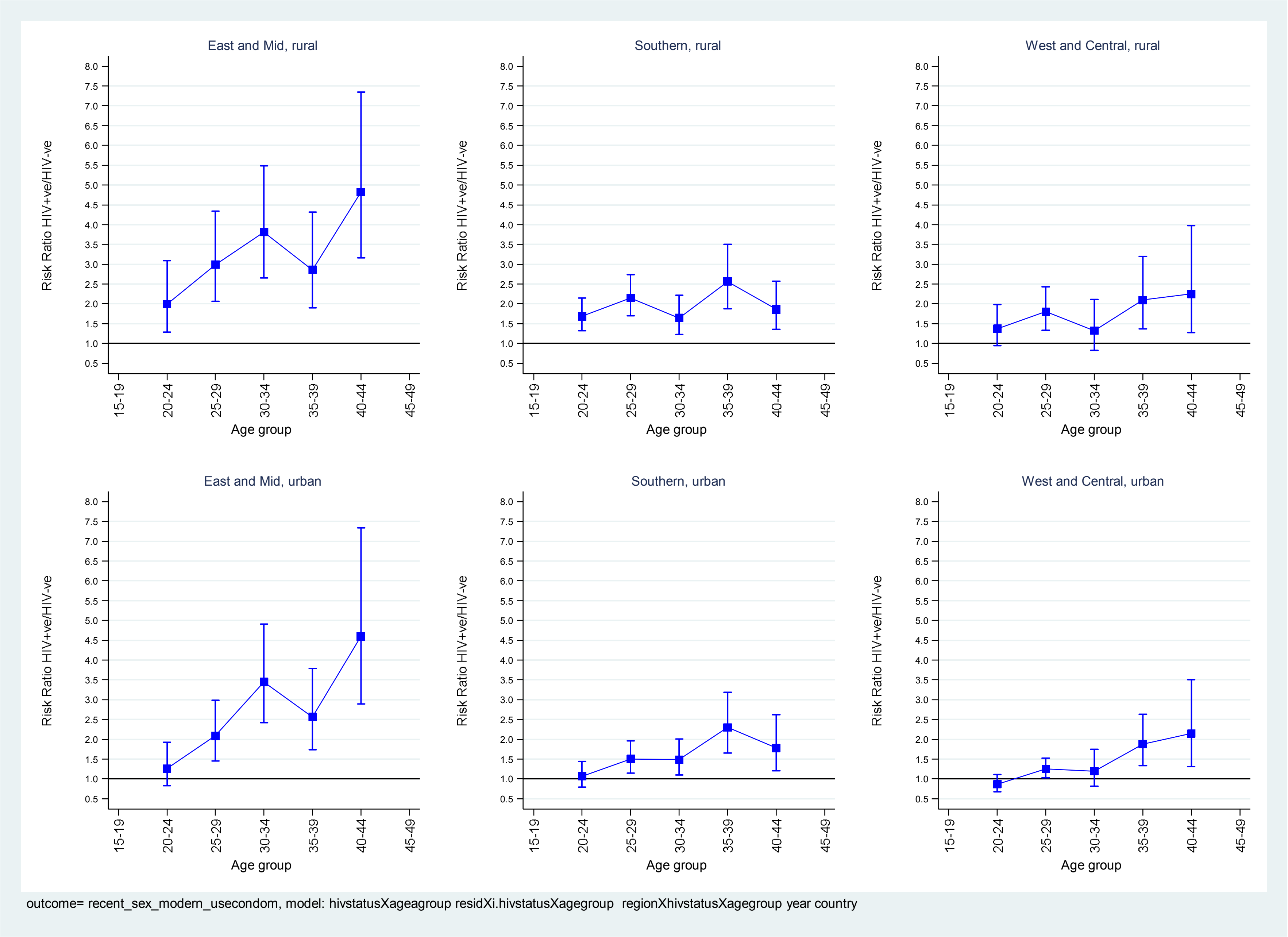

RECENT EXPOSURE TO PREGNANCY

Table E: Risk ratios of recent exposure to a live birth, using log binomial model

	Model 1	Model 2	Model 3	Model 4
	FRR 95 %CI	FRR 95 %CI	FRR 95 %CI	FRR 95 %CI
HIV status				
HIV negative	1	1	1	1
HIV Positive	0.90(0.83-0.97)	1.01(0.89-1.14)	1.01(0.89-1.15)	1.00(0.88-1.15)
Effects of HIV by age				
15-19, HIV positive	1.85(1.52-2.24)	1.78(1.24-2.56)	1.79(1.25-2.58)	1.79(1.25-2.58)
20-24, HIV positive	1.29(1.15-1.44)	1.38(1.16-1.65)	1.37(1.15-1.64)	1.38(1.15-1.64)
25-29, HIV positive	1.12(1.01-1.24)	1.23(1.05-1.44)	1.23(1.05-1.44)	1.23(1.05-1.44)
30-34, HIV positive	1	1	1	1
35-39, HIV positive	0.86(0.76-0.96)	0.79(0.66-0.94)	0.79(0.66-0.94)	0.79(0.66-0.94)
40-44, HIV positive	0.90(0.80-1.01)	0.81(0.66-0.98)	0.82(0.67-0.99)	0.82(0.67-0.99)
45-49, HIV positive	0.84(0.73-0.96)	0.73(0.57-0.94)	0.74(0.58-0.94)	0.74(0.58-0.94)
Effects of HIV by Place of residence				
rural, HIV positive		0.93(0.80-1.08)	0.92(0.79-1.07)	0.92(0.79-1.07)
Effects of Place of residence on age and HIV status interaction				
rural, HIV positive,15-19		1.06(0.69-1.63)	1.07(0.70-1.64)	1.07(0.70-1.64)
rural, HIV positive,20-24		0.90(0.72-1.13)	0.91(0.73-1.14)	0.91(0.73-1.14)
rural, HIV positive,25-29		0.85(0.69-1.05)	0.84(0.68-1.04)	0.84(0.68-1.04)
rural, HIV positive,30-34		1	1	1
rural, HIV positive,35-39		1.16(0.92-1.46)	1.16(0.92-1.46)	1.16(0.92-1.46)
rural, HIV positive,40-44		1.19(0.93-1.53)	1.18(0.92-1.52)	1.18(0.92-1.52)
rural, HIV positive,45-49		1.23(0.92-1.65)	1.22(0.91-1.64)	1.22(0.91-1.64)
Effects of HIV by Region				
Southern, HIV positive		0.95(0.87-1.04)	0.95(0.87-1.04)	0.95(0.87-1.05)
Eastern, HIV positive		1	1	1
Western, HIV positive		0.93(0.85-1.01)	0.93(0.86-1.01)	0.93(0.85-1.02)
Effects of HIV by ART Coverage				
<20%, HIV positive				1
20-49%, HIV positive				1.02(0.94-1.10)
>50%, HIV positive				1.02(0.92-1.13)
Age Group				
15-19	0.33(0.32-0.34)	0.28(0.26-0.31)	0.28(0.26-0.30)	0.28(0.26-0.30)
20-24	0.75(0.73-0.77)	0.66(0.62-0.70)	0.66(0.62-0.70)	0.66(0.62-0.70)
25-29	0.94(0.91-0.96)	0.87(0.83-0.92)	0.87(0.83-0.92)	0.87(0.83-0.92)
30-34	1			
35-39	1.07(1.04-1.10)	1.09(1.03-1.15)	1.09(1.03-1.15)	1.09(1.03-1.15)
40-44	1.12(1.09-1.15)	1.14(1.07-1.20)	1.12(1.06-1.19)	1.12(1.06-1.19)
45-49	1.12(1.09-1.15)	1.12(1.05-1.19)	1.13(1.06-1.20)	1.13(1.06-1.20)
Place of residence				
urban		1	1	1
rural		1.21(1.16-1.27)	1.23(1.18-1.29)	1.23(1.18-1.29)
Effects of age by Place of residence				
rural, 15-19		1.30(1.19-1.42)	1.29(1.18-1.41)	1.29(1.18-1.41)
rural, 20-24		1.24(1.17-1.32)	1.24(1.16-1.32)	1.24(1.16-1.32)
rural, 25-29		1.12(1.05-1.19)	1.12(1.05-1.19)	1.12(1.05-1.19)
rural, 30-34		1	1	1
rural, 35-39		0.98(0.92-1.04)	0.97(0.91-1.03)	0.97(0.91-1.03)
rural, 40-44		0.97(0.91-1.04)	0.98(0.92-1.04)	0.98(0.92-1.04)
rural, 45-49		0.98(0.92-1.05)	0.97(0.91-1.04)	0.97(0.91-1.04)
ART Coverage				
<20%				1
20-49%			0.87(0.78-0.98)	0.87(0.78-0.98)
>50%			0.79(0.68-0.91)	0.78(0.67-0.91)

Table F: Stratum specific risk ratios from Model 1, exposure to live birth

		Southern Africa	East and Mid	West and central
Urban				
	15-19	1.70 (1.21-2.40)	1.79 (1.27-2.52)	1.67 (1.19-2.33)
	20-24	1.32 (1.13-1.55)	1.39 (1.20-1.62)	1.30 (1.12-1.50)
	25-29	1.18 (1.03-1.35)	1.24 (1.09-1.41)	1.15 (1.02-1.30)
	30-34	0.96 (0.84-1.09)	1.01 (0.89-1.14)	0.94 (0.83-1.06)
	35-39	0.75 (0.65-0.88)	0.79 (0.69-0.92)	0.74 (0.64-0.85)
	40-44	0.77 (0.64-0.93)	0.81 (0.68-0.96)	0.75 (0.64-0.89)
	45-49	0.70 (0.55-0.89)	0.74 (0.58-0.93)	0.68 (0.54-0.86)
Rural				
	15-19	1.68 (1.37-2.07)	1.77 (1.44-2.17)	1.64 (1.34-2.02)
	20-24	1.11 (0.98-1.25)	1.17 (1.04-1.31)	1.09 (0.97-1.22)
	25-29	0.93 (0.83-1.04)	0.98 (0.87-1.09)	0.91 (0.81-1.02)
	30-34	0.89 (0.79-0.99)	0.93 (0.84-1.04)	0.87 (0.77-0.97)
	35-39	0.81 (0.72-0.92)	0.85 (0.76-0.96)	0.80 (0.70-0.90)
	40-44	0.85 (0.75-0.97)	0.90 (0.79-1.02)	0.83 (0.73-0.95)
	45-49	0.80 (0.69-0.92)	0.84 (0.73-0.96)	0.78 (0.68-0.90)

Figure E: Risk ratio of exposure to pregnancy, comparing HIV positive women to HIV negative women

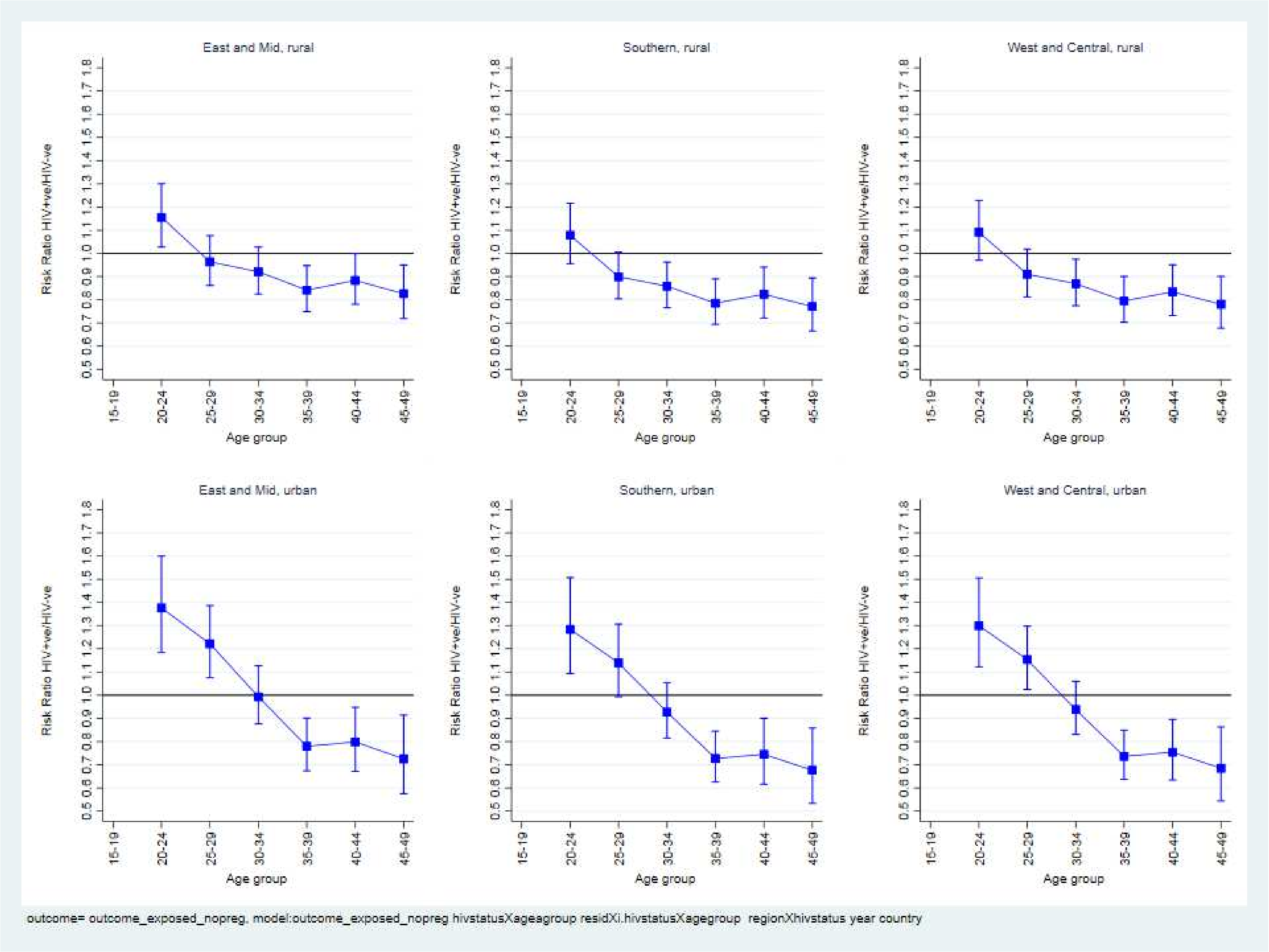

Figure F: Adjusted Risk ratio for recent sex and exposure to pregnancy, comparing HIV positive women to negative women with Fertility rate ratio

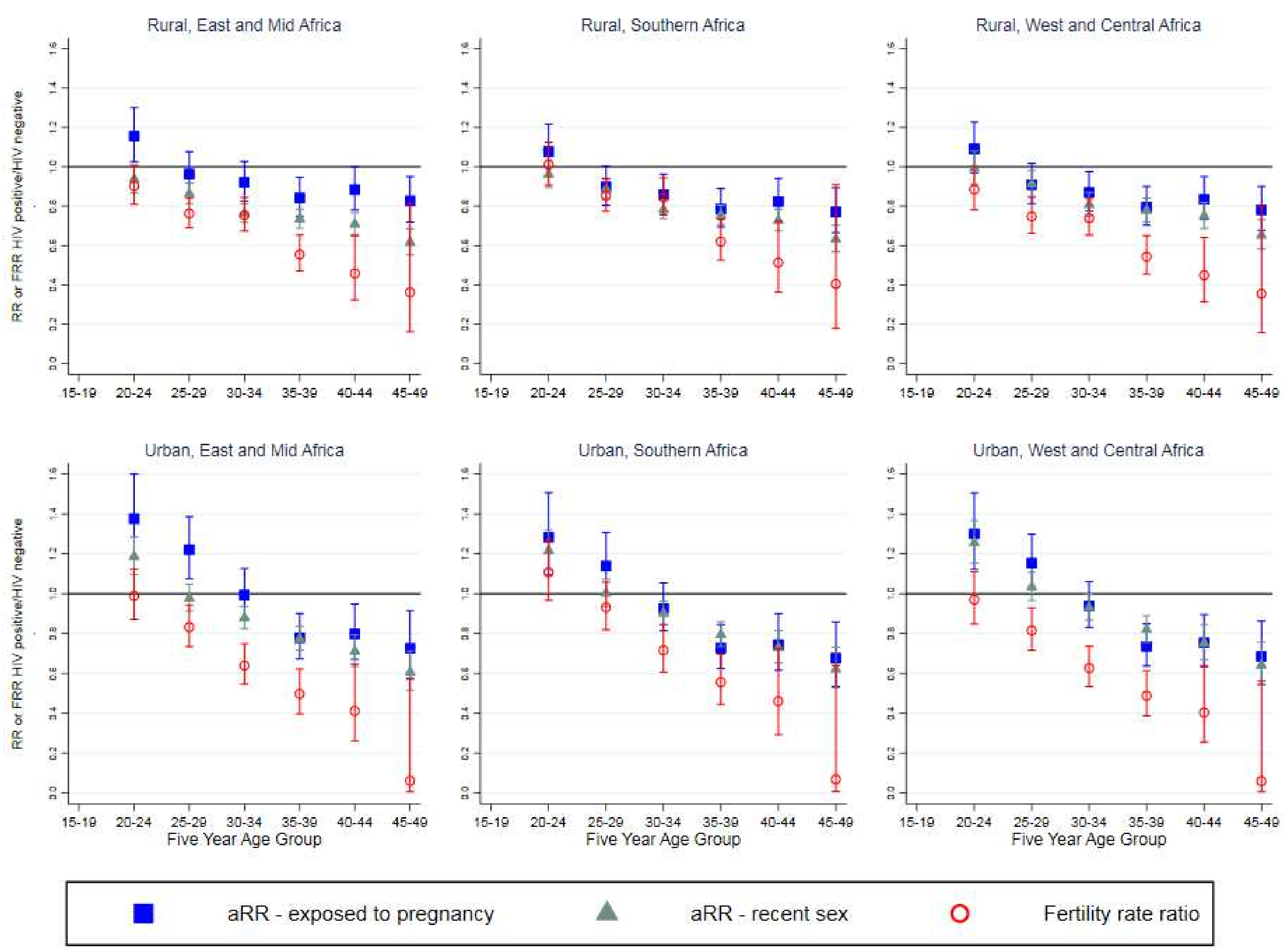

MARRIED

Table G: Risk ratios of being married using Log Binomial model

	Model 1		Model 2		Model 3	
Married - All women	RR	95 %CI	RR	95 %CI	RR	95 %CI
HIV status						
HIV negative	1		1		1	
HIV Positive	0.79	(0.76-0.81)	0.78	(0.74-0.83)	0.76	(0.72-0.81)
Effects of HIV by age						
15-19, HIV positive	1.77	(1.59-1.98)	2.16	(1.74-2.68)	2.17	(1.75-2.69)
20-24, HIV positive	1.16	(1.10-1.23)	1.36	(1.24-1.49)	1.37	(1.25-1.50)
25-29, HIV positive	1.08	(1.04-1.13)	1.18	(1.10-1.26)	1.18	(1.11-1.27)
30-34, HIV positive	1		1		1	
35-39, HIV positive	0.93	(0.89-0.98)	0.89	(0.82-0.97)	0.89	(0.82-0.97)
40-44, HIV positive	0.86	(0.81-0.91)	0.84	(0.76-0.94)	0.85	(0.76-0.94)
45-49, HIV positive	0.77	(0.72-0.83)	0.74	(0.65-0.85)	0.75	(0.66-0.86)
Effects of HIV by Place of residence						
rural, HIV positive			1.02	(0.95-1.08)	1.02	(0.96-1.08)
Effects of Place of residence on age and HIV status interaction						
rural, HIV positive,15-19			0.77	(0.60-0.98)	0.77	(0.60-0.99)
rural, HIV positive,20-24			0.80	(0.72-0.89)	0.80	(0.72-0.89)
rural, HIV positive,25-29			0.88	(0.81-0.96)	0.88	(0.81-0.95)
rural, HIV positive,30-34			1		1	
rural, HIV positive,35-39			1.06	(0.96-1.17)	1.06	(0.96-1.16)
rural, HIV positive,40-44			1.03	(0.91-1.16)	1.02	(0.90-1.15)
rural, HIV positive,45-49			1.03	(0.88-1.21)	1.02	(0.87-1.19)
Effects of HIV by Region						
Southern, HIV positive			1.00	(0.96-1.03)	1.00	(0.96-1.03)
Western, HIV positive			1.07	(1.03-1.12)	1.08	(1.04-1.13)
Effects of HIV by ART Coverage						
<20%, HIV positive					1	
20-49%, HIV positive					1.03	(0.99-1.06)
>50%, HIV positive					1.06	(1.02-1.11)
Age Group						
15-19	0.27	(0.26-0.27)	0.18	(0.17-0.19)	0.18	(0.17-0.19)
20-24	0.76	(0.75-0.77)	0.60	(0.59-0.62)	0.60	(0.58-0.62)
25-29	0.96	(0.96-0.97)	0.90	(0.88-0.92)	0.90	(0.88-0.91)
30-34	1		1		1	
35-39	1.01	(1.00-1.01)	1.04	(1.02-1.06)	1.04	(1.02-1.06)
40-44	0.98	(0.97-0.99)	1.00	(0.98-1.02)	1.00	(0.98-1.02)
45-49	0.94	(0.93-0.94)	0.94	(0.91-0.96)	0.94	(0.91-0.96)
Place of residence						
urban			1		1	
rural			1.11	(1.10-1.13)	1.11	(1.10-1.13)
Effects of age by Place of residence						
rural, 15-19			1.77	(1.65-1.90)	1.77	(1.65-1.90)
rural, 20-24			1.42	(1.38-1.46)	1.43	(1.39-1.47)
rural, 25-29			1.10	(1.08-1.12)	1.11	(1.08-1.13)
rural, 30-34			1		1	
rural, 35-39			0.96	(0.94-0.98)	0.96	(0.94-0.98)
rural, 40-44			0.97	(0.95-0.99)	0.97	(0.95-1.00)
rural, 45-49			1.00	(0.97-1.03)	1.00	(0.97-1.03)
ART Coverage						
<20%					1	
20-49%					1.02	(0.99-1.05)
>50%					1.03	(0.98-1.09)

Table H: Stratum specific risk ratios of being married comparing HIV positive women to HIV negative women.

		Risk ratios by region		
		Southern Africa	East and Mid	West and central
Urban				
	15-19	1.67 (1.35-2.08)	1.69 (1.36-2.11)	1.81 (1.46-2.26)
	20-24	1.07 (0.98-1.17)	1.09 (1.00-1.18)	1.16 (1.07-1.27)
	25-29	0.92 (0.87-0.97)	0.94 (0.89-0.99)	1.00 (0.95-1.05)
	30-34	0.78 (0.74-0.83)	0.79 (0.75-0.84)	0.85 (0.80-0.90)
	35-39	0.70 (0.65-0.75)	0.71 (0.66-0.76)	0.76 (0.71-0.81)
	40-44	0.66 (0.59-0.72)	0.66 (0.60-0.73)	0.71 (0.65-0.78)
	45-49	0.57 (0.49-0.65)	0.58 (0.50-0.66)	0.62 (0.54-0.71)
Rural				
	15-19	1.28 (1.13-1.46)	1.30 (1.14-1.48)	1.39 (1.22-1.59)
	20-24	0.85 (0.80-0.90)	0.86 (0.81-0.91)	0.92 (0.87-0.97)
	25-29	0.83 (0.79-0.86)	0.84 (0.80-0.87)	0.90 (0.86-0.94)
	30-34	0.79 (0.76-0.82)	0.80 (0.77-0.83)	0.86 (0.82-0.90)
	35-39	0.75 (0.71-0.78)	0.76 (0.72-0.80)	0.81 (0.77-0.86)
	40-44	0.69 (0.65-0.73)	0.70 (0.65-0.74)	0.74 (0.70-0.80)
	45-49	0.61 (0.56-0.66)	0.62 (0.57-0.67)	0.66 (0.61-0.72)

Figure G: Adjusted Risk ratio for being married, comparing HIV positive women to negative women.

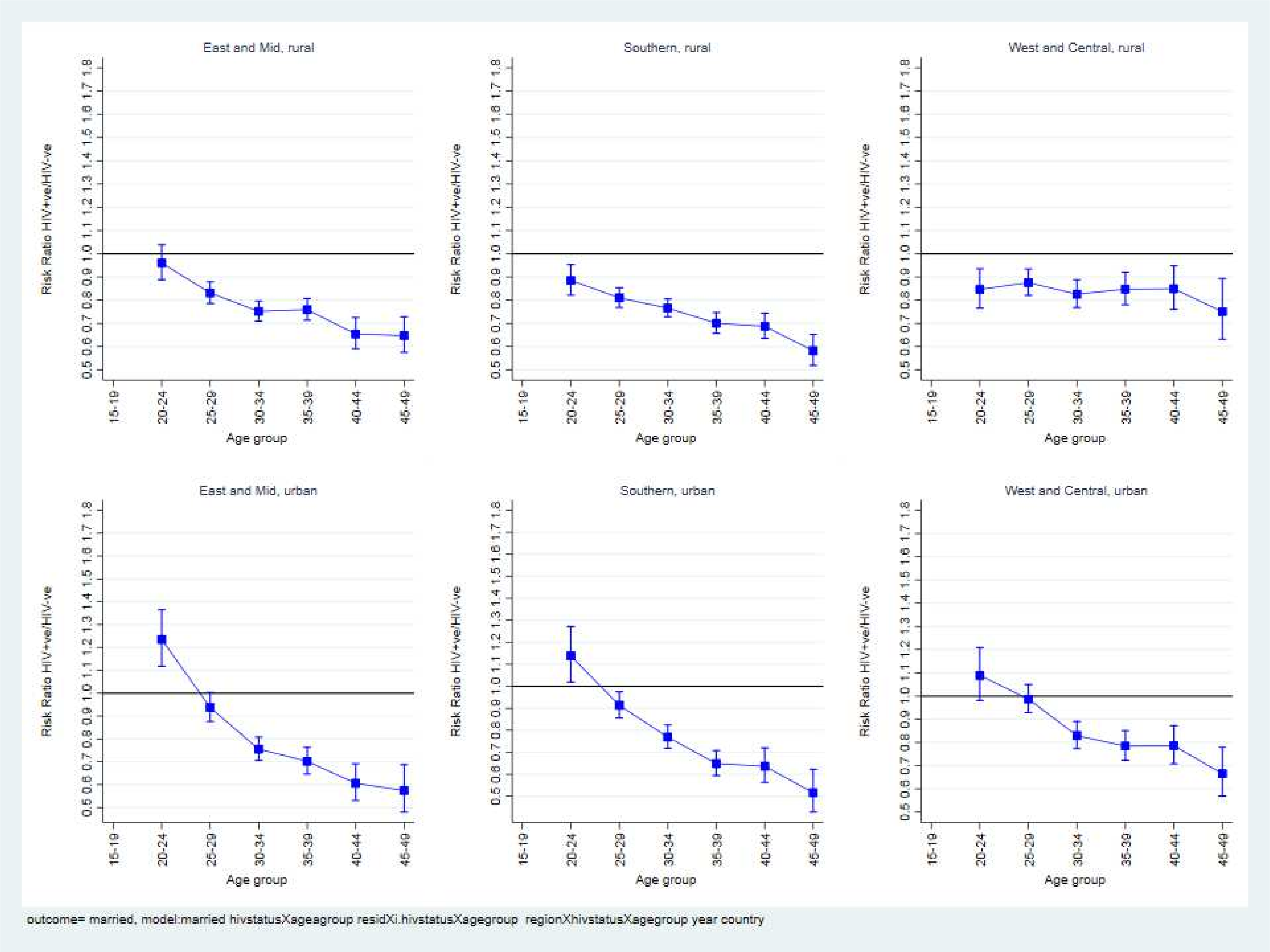

MARRIED RECENT SEX AND EXPOSURE TO PREGNANCY

Table I: Risk ratios of recent sex among ***married women****, using log binomial model*

	Model 1		Model 2		Model 3		Model 4	
	RR	95 %CI	RR	95 %CI	RR	95 %CI	RR	95 %CI
HIV status								
HIV negative	1		1		1		1	
HIV Positive	0.98	(0.95-1.01)	0.99	(0.96-1.03)	0.99	(0.95-1.03)	1.03	(0.98-1.08)
Effects of HIV by age								
15-19, HIV positive	1.10	(1.00-1.22)	1.10	(0.99-1.21)	1.10	(1.00-1.22)	0.94	(0.79-1.13)
20-24, HIV positive	1.06	(1.01-1.12)	1.07	(1.01-1.12)	1.07	(1.01-1.13)	1.04	(0.97-1.12)
25-29, HIV positive	1.03	(0.99-1.08)	1.03	(0.99-1.08)	1.03	(0.99-1.08)	1.00	(0.95-1.06)
30-34, HIV positive	1		1		1		1	
35-39, HIV positive	0.97	(0.93-1.02)	0.97	(0.93-1.02)	0.98	(0.93-1.02)	0.95	(0.90-1.01)
40-44, HIV positive	0.98	(0.93-1.03)	0.98	(0.93-1.03)	0.98	(0.93-1.03)	0.95	(0.88-1.02)
45-49, HIV positive	0.96	(0.90-1.04)	0.97	(0.90-1.04)	0.96	(0.90-1.04)	0.92	(0.81-1.03)
Effects of HIV by Place of residence								
rural, HIV positive			0.95	(0.92-0.98)	0.95	(0.92-0.98)	0.90	(0.85-0.96)
Effects of Place of residence on age and HIV status interaction								
rural, HIV positive,15-19							1.32	(1.07-1.62)
rural, HIV positive,20-24							1.03	(0.94-1.14)
rural, HIV positive,25-29							1.06	(0.98-1.16)
rural, HIV positive,30-34							1	
rural, HIV positive,35-39							1.06	(0.97-1.16)
rural, HIV positive,40-44							1.10	(0.99-1.22)
rural, HIV positive,45-49							1.11	(0.95-1.29)
Effects of HIV by Region								
Southern, HIV positive			1.01	(0.98-1.04)	1.01	(0.98-1.04)	1.00	(0.97-1.04)
Western, HIV positive			1.00	(0.96-1.05)	1.01	(0.96-1.05)	0.99	(0.95-1.04)
Effects of HIV by ART Coverage								
20-49%, HIV positive							0.99	(0.95-1.03)
>50%, HIV positive							0.97	(0.93-1.01)
Age Group								
15-19	0.87	(0.85-0.89)	0.88	(0.85-0.90)	0.87	(0.85-0.89)	0.91	(0.87-0.95)
20-24	0.95	(0.94-0.97)	0.95	(0.94-0.97)	0.95	(0.94-0.96)	0.97	(0.95-1.00)
25-29	0.99	(0.97-1.00)	0.99	(0.97-1.00)	0.99	(0.97-1.00)	0.99	(0.97-1.01)
30-34	1.00	(1.00-1.00)	1.00	(1.00-1.00)	1.00	(1.00-1.00)	1.00	(1.00-1.00)
35-39	1.01	(1.00-1.02)	1.01	(1.00-1.02)	1.01	(0.99-1.02)	1.00	(0.98-1.03)
40-44	1.01	(0.99-1.02)	1.01	(0.99-1.02)	1.01	(0.99-1.02)	0.98	(0.96-1.01)
45-49	0.98	(0.96-1.00)	0.98	(0.96-1.00)	0.98	(0.97-1.00)	0.98	(0.95-1.01)
Place of residence								
urban			1		1		1	
rural			0.92	(0.91-0.93)	0.91	(0.90-0.93)	0.92	(0.90-0.95)
Effects of age by Place of residence								
rural, 15-19							0.94	(0.89-0.99)
rural, 20-24							0.96	(0.94-0.99)
rural, 25-29							1.00	(0.97-1.02)
rural, 30-34							1	
rural, 35-39							1.01	(0.98-1.04)
rural, 40-44							1.03	(1.00-1.07)
rural, 45-49							1.01	(0.97-1.04)
ART Coverage								
<20%					1		1	
20-49%					0.99	(0.93-1.06)	1.00	(0.94-1.06)
>50%					0.99	(0.91-1.08)	1.00	(0.92-1.09)

*Table J: Risk ratios of exposure to a live birth, among **married women,**, using log binomial model*

	Model 1		Model 2		Model 3		Model 4	
	RR	95 %CI	RR	95 %CI	RR	95 %CI	RR	95 %CI
HIV status								
HIV negative	1		1		1		1	
HIV Positive	1.07(1.00-1.15)		1.17(1.07-1.29)		1.18(1.07-1.30)		1.22(1.08-1.39)	
Effects of HIV by age								
15-19, HIV positive	1.16(0.98-1.36)		1.18(1.00-1.40)		1.18(1.00-1.40)		1.03(0.76-1.41)	
20-24, HIV positive	1.07(0.97-1.20)		1.08(0.97-1.20)		1.07(0.96-1.19)		1.02(0.86-1.20)	
25-29, HIV positive	0.99(0.90-1.09)		0.99(0.89-1.09)		0.98(0.89-1.08)		1.03(0.89-1.20)	
30-34, HIV positive	1		1		1		1	
35-39, HIV positive	0.87(0.78-0.97)		0.87(0.78-0.97)		0.87(0.78-0.97)		0.82(0.69-0.97)	
40-44, HIV positive	0.97(0.87-1.08)		0.97(0.87-1.08)		0.98(0.88-1.09)		0.92(0.77-1.10)	
45-49, HIV positive	0.97(0.86-1.10)		0.97(0.86-1.10)		0.98(0.87-1.11)		0.94(0.74-1.19)	
Effects of HIV by Place of residence								
rural, HIV positive			0.99(0.92-1.05)		0.98(0.92-1.05)		0.93(0.81-1.08)	
Effects of Place of residence on age and HIV status interaction								
rural, HIV positive,15-19							1.22(0.86-1.72)	
rural, HIV positive,20-24							1.09(0.88-1.34)	
rural, HIV positive,25-29							0.92(0.76-1.12)	
rural, HIV positive,30-34							1.00(1.00-1.00)	
rural, HIV positive,35-39							1.12(0.91-1.39)	
rural, HIV positive,40-44							1.11(0.89-1.38)	
rural, HIV positive,45-49							1.07(0.82-1.40)	
Effects of HIV by Region								
Southern, HIV positive			0.93(0.86-1.02)		0.93(0.86-1.02)		0.93(0.86-1.02)	
Western, HIV positive			0.89(0.82-0.96)		0.89(0.83-0.96)		0.89(0.82-0.96)	
Effects of HIV by ART Coverage								
20-49%, HIV positive							0.98(0.92-1.06)	
>50%, HIV positive							0.98(0.90-1.08)	
Age Group								
15-19	0.99(0.95-1.02)		0.98(0.95-1.02)		0.97(0.94-1.01)		1.00(0.93-1.08)	
20-24	0.96(0.93-0.98)		0.96(0.93-0.98)		0.96(0.93-0.98)		0.95(0.90-1.00)	
25-29	0.98(0.96-1.00)		0.98(0.96-1.01)		0.98(0.96-1.01)		0.96(0.91-1.01)	
30-34	1		1		1		1	
35-39	1.07(1.04-1.09)		1.07(1.04-1.09)		1.07(1.04-1.09)		1.08(1.02-1.14)	
40-44	1.15(1.12-1.18)		1.14(1.12-1.17)		1.14(1.11-1.17)		1.14(1.08-1.21)	
45-49	1.21(1.18-1.24)		1.20(1.17-1.23)		1.20(1.17-1.23)		1.23(1.16-1.30)	
Place of residence								
urban			1		1		1	
rural			1.12(1.10-1.14)		1.13(1.11-1.15)		1.13(1.08-1.18)	
Effects of age by Place of residence								
rural, 15-19							0.97(0.89-1.05)	
rural, 20-24							1.00(0.95-1.06)	
rural, 25-29							1.03(0.98-1.09)	
rural, 30-34							1	
rural, 35-39							0.99(0.93-1.05)	
rural, 40-44							0.99(0.93-1.06)	
rural, 45-49							0.97(0.91-1.03)	
ART Coverage								
<20%								
20-49%					0.89(0.80-0.99)		0.89(0.80-0.99)	
>50%					0.76	(0.66-0.87)	0.77(0.67-0.88)	

*Table K: Stratum specific risk ratios, recent sex, **married women**, exclude pregnancy*

		Region		
		Southern Africa	East and Mid	West and central
Urban				
	15-19	1.10 (0.99-1.21)	1.09 (0.98-1.21)	1.09 (0.99-1.21)
	20-24	1.07 (1.02-1.12)	1.06 (1.01-1.11)	1.06 (1.01-1.13)
	25-29	1.03 (0.99-1.08)	1.02 (0.99-1.06)	1.03 (0.98-1.08)
	30-34	1.00 (0.96-1.04)	0.99 (0.96-1.03)	1.00 (0.95-1.05)
	35-39	0.97 (0.93-1.01)	0.96 (0.93-1.00)	0.97 (0.92-1.02)
	40-44	0.98 (0.93-1.03)	0.97 (0.93-1.02)	0.98 (0.92-1.03)
	45-49	0.97 (0.90-1.04)	0.96 (0.90-1.03)	0.96 (0.89-1.05)
Rural				
	15-19	1.04 (0.94-1.15)	1.03 (0.93-1.14)	1.04 (0.94-1.15)
	20-24	1.01 (0.97-1.06)	1.00 (0.96-1.05)	1.01 (0.95-1.07)
	25-29	0.98 (0.94-1.02)	0.97 (0.93-1.01)	0.98 (0.93-1.03)
	30-34	0.95 (0.91-0.99)	0.94 (0.91-0.98)	0.95 (0.90-1.00)
	35-39	0.92 (0.88-0.96)	0.91 (0.88-0.95)	0.92 (0.87-0.97)
	40-44	0.93 (0.89-0.97)	0.92 (0.88-0.97)	0.93 (0.87-0.98)
	45-49	0.92 (0.86-0.98)	0.91 (0.85-0.98)	0.91 (0.84-0.99)

*Table L: Stratum specific risk ratios, Exposed, **married women**, exclude pregnancy*

		Region		
		Southern Africa	East and Mid	West and central
Urban				
	15-19	1.29 (1.09-1.54)	1.39 (1.18-1.63)	1.23 (1.06-1.43)
	20-24	1.18 (1.05-1.32)	1.27 (1.14-1.40)	1.12 (1.02-1.24)
	25-29	1.08 (0.97-1.21)	1.16 (1.06-1.27)	1.03 (0.94-1.13)
	30-34	1.10 (0.99-1.22)	1.17 (1.07-1.29)	1.04 (0.95-1.15)
	35-39	0.96 (0.86-1.07)	1.03 (0.93-1.13)	0.91 (0.83-1.00)
	40-44	1.06 (0.94-1.20)	1.14 (1.03-1.26)	1.01 (0.92-1.12)
	45-49	1.07 (0.93-1.22)	1.14 (1.01-1.29)	1.01 (0.90-1.15)
Rural				
	15-19	1.28 (1.08-1.51)	1.37 (1.16-1.61)	1.21 (1.04-1.41)
	20-24	1.17 (1.05-1.29)	1.25 (1.13-1.37)	1.11 (1.01-1.22)
	25-29	1.07 (0.97-1.18)	1.15 (1.05-1.25)	1.02 (0.93-1.11)
	30-34	1.08 (0.99-1.18)	1.16 (1.06-1.26)	1.03 (0.94-1.13)
	35-39	0.94 (0.85-1.05)	1.01 (0.92-1.11)	0.90 (0.81-0.99)
	40-44	1.05 (0.94-1.17)	1.12 (1.02-1.24)	1.00 (0.91-1.10)
	45-49	1.05 (0.93-1.19)	1.13 (1.01-1.26)	1.00 (0.89-1.12)

*Figure H: Adjusted Risk ratio for recent sex among **married women**, comparing HIV positive women to HIV negative women.*

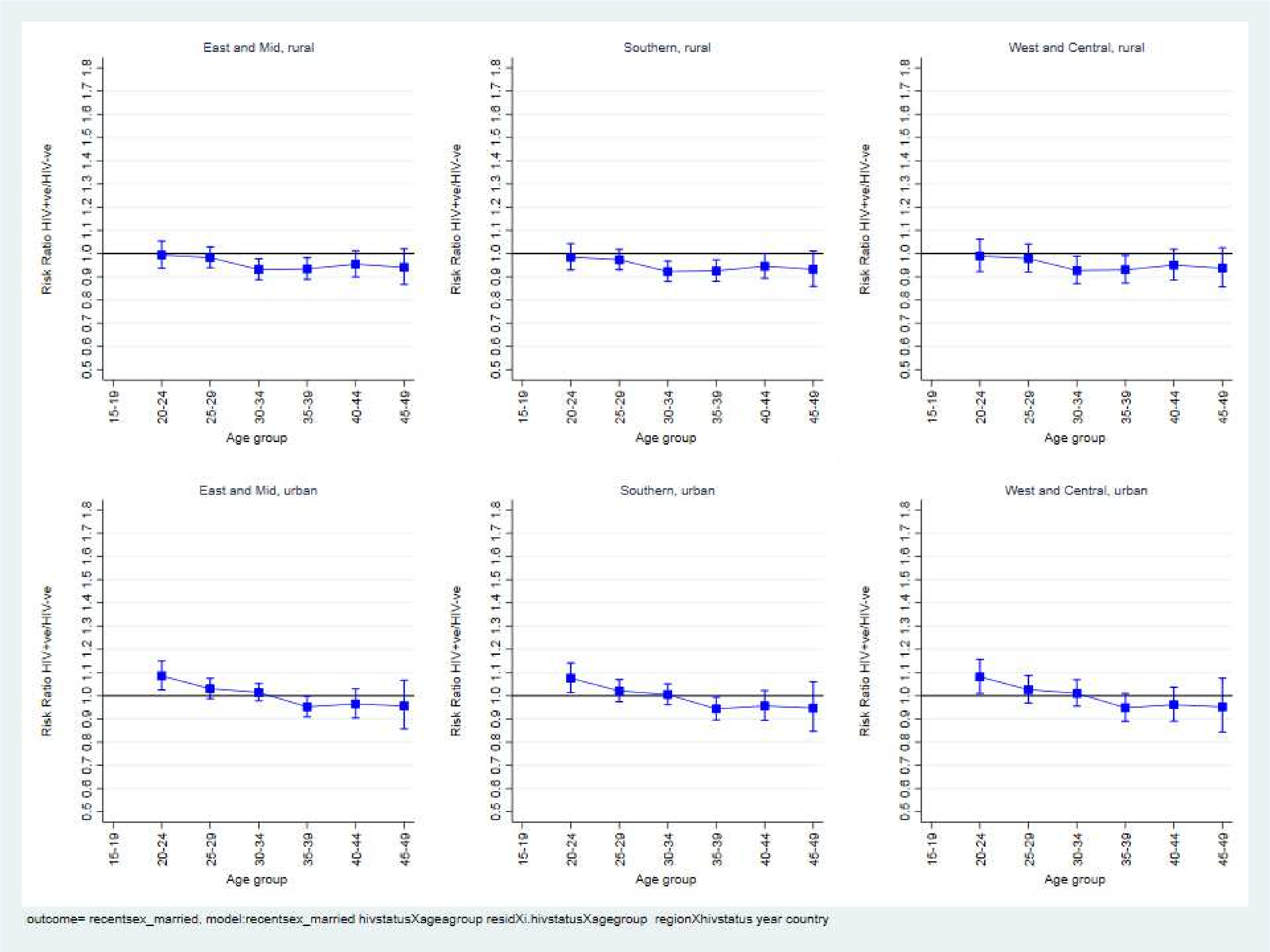

*Figure I: Risk ratio of currently using modern contraceptives among **married women** reporting recent sex, comparing HIV positive women to HIV negative women*

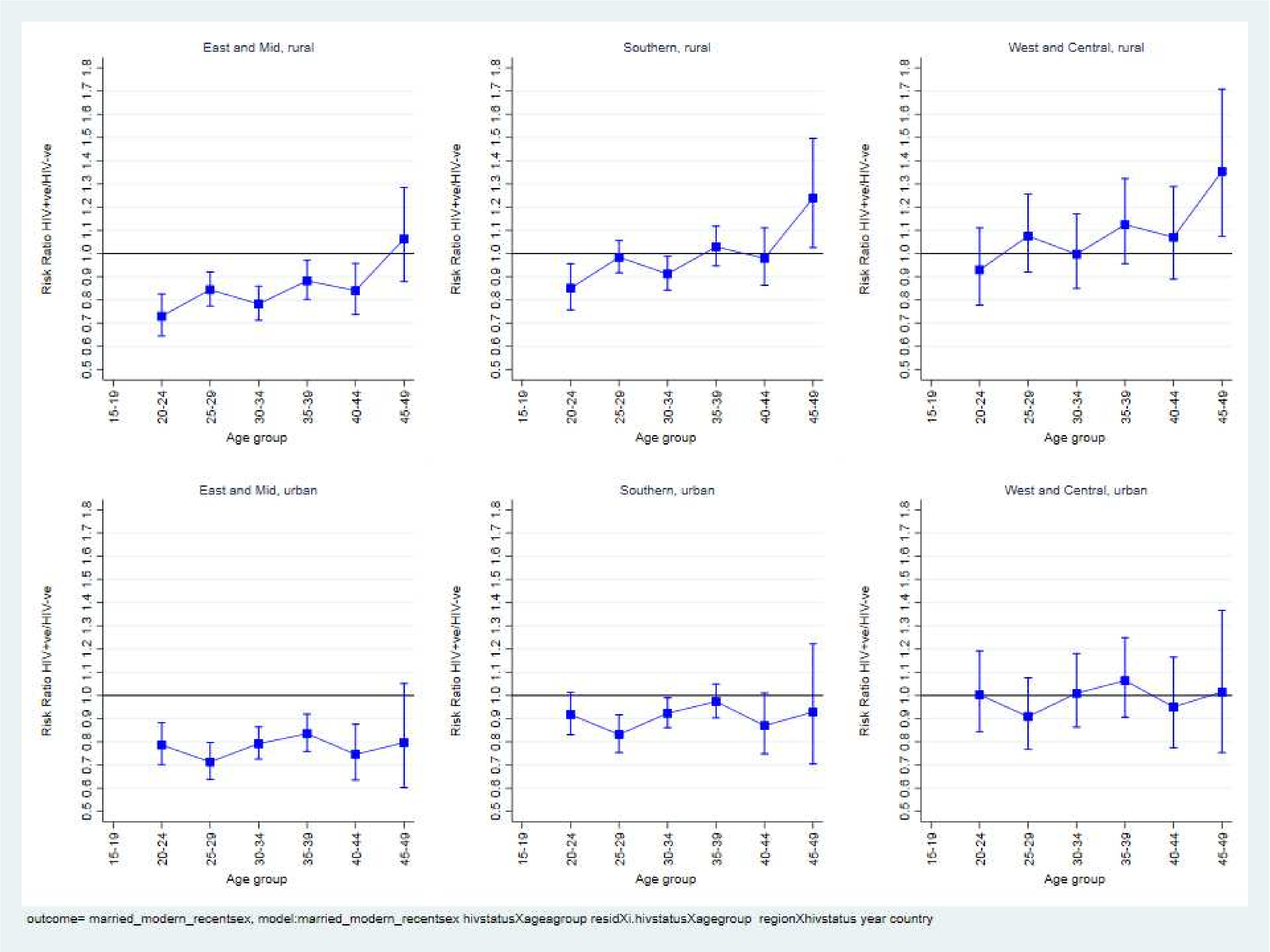

Figure J: Adjusted Risk ratio for exposure to pregnancy among ***married women****, comparing HIV positive women to negative women.*

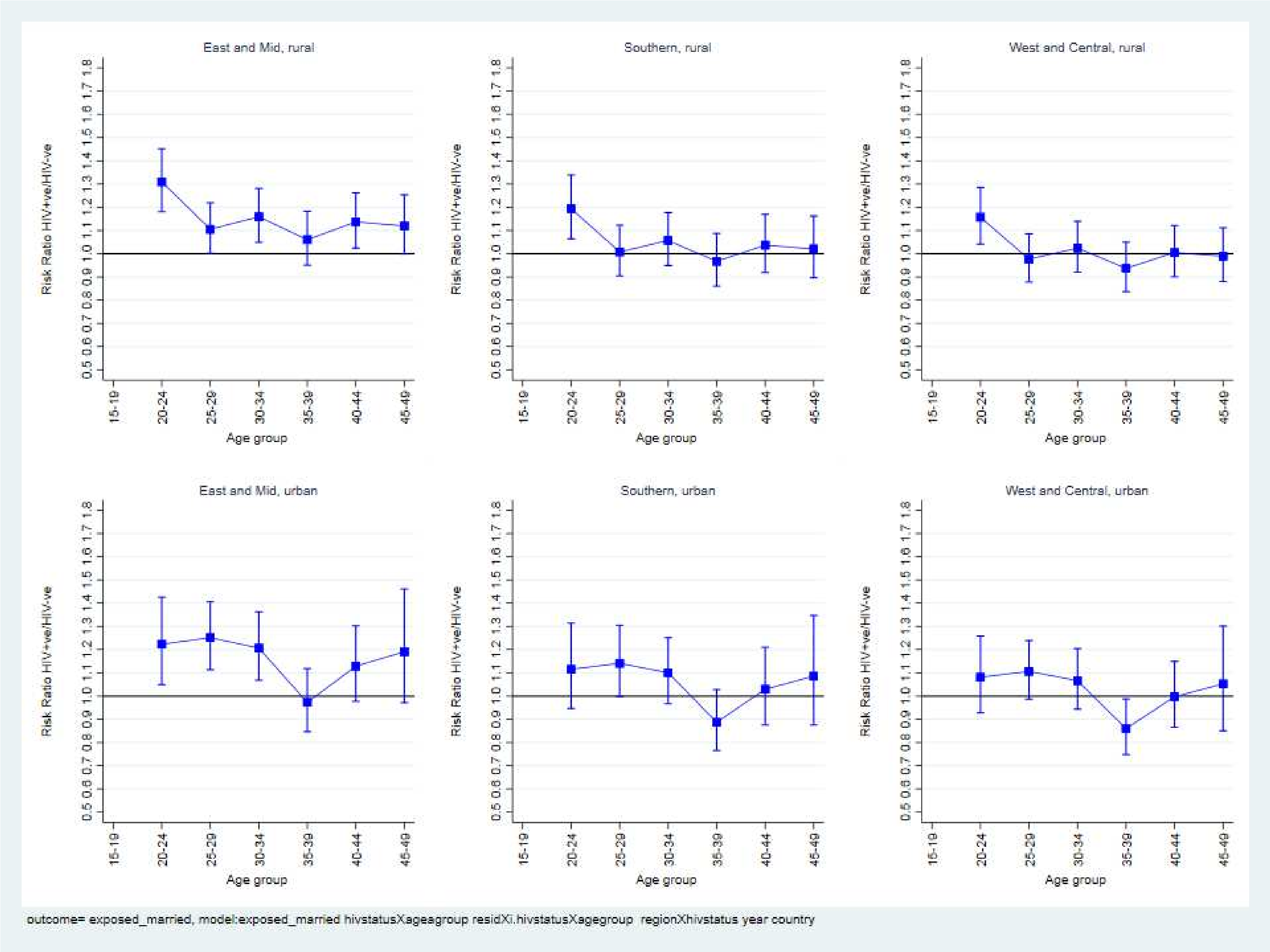

Figure K: Adjusted Risk ratio for recent sex and exposure to a live birth, comparing HIV positive women to negative women with Fertility rate ratio for ***married women***

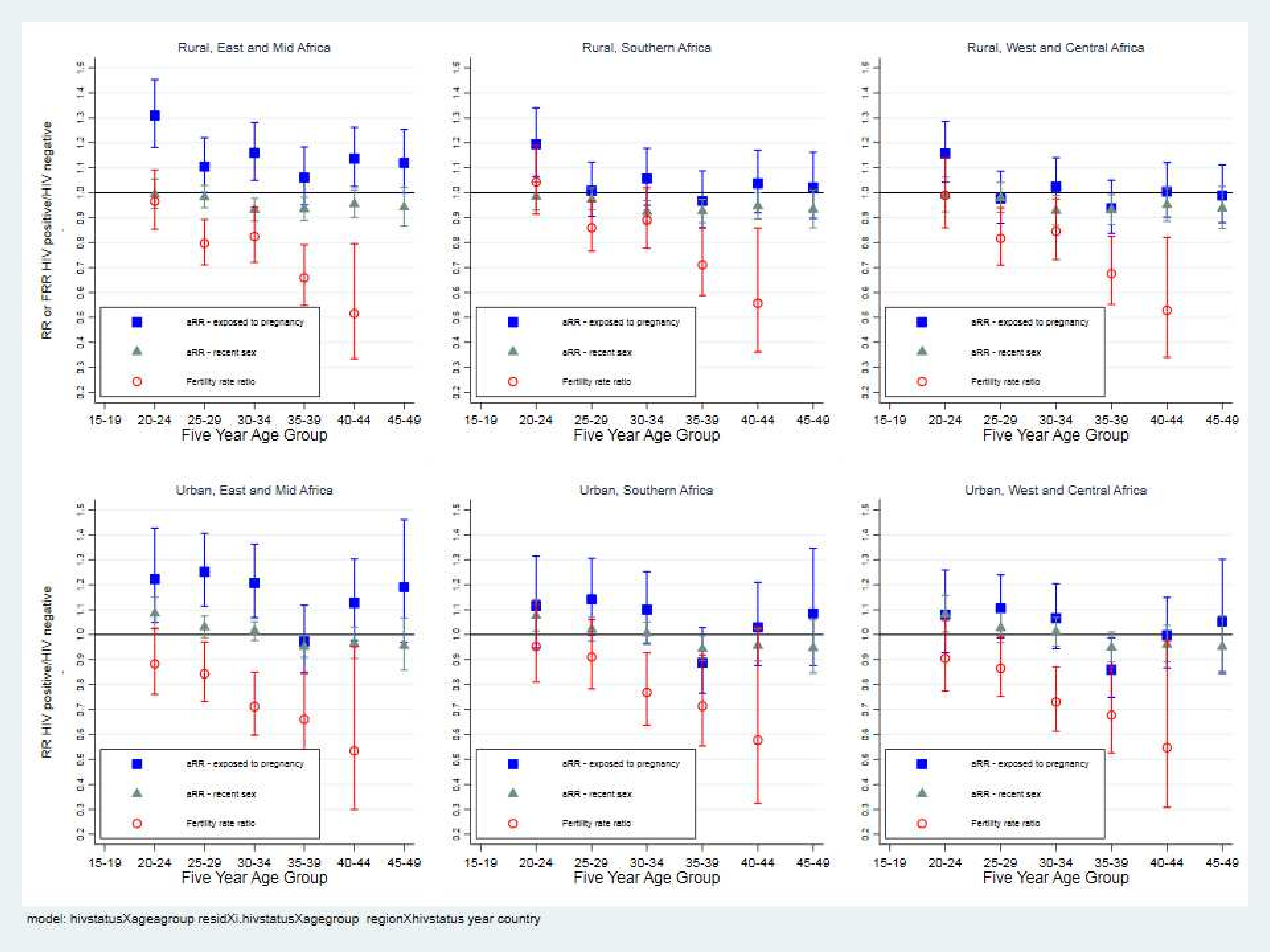

*Figure L: Adjusted Fertility rate ratio (FRR/RR) for **married women***

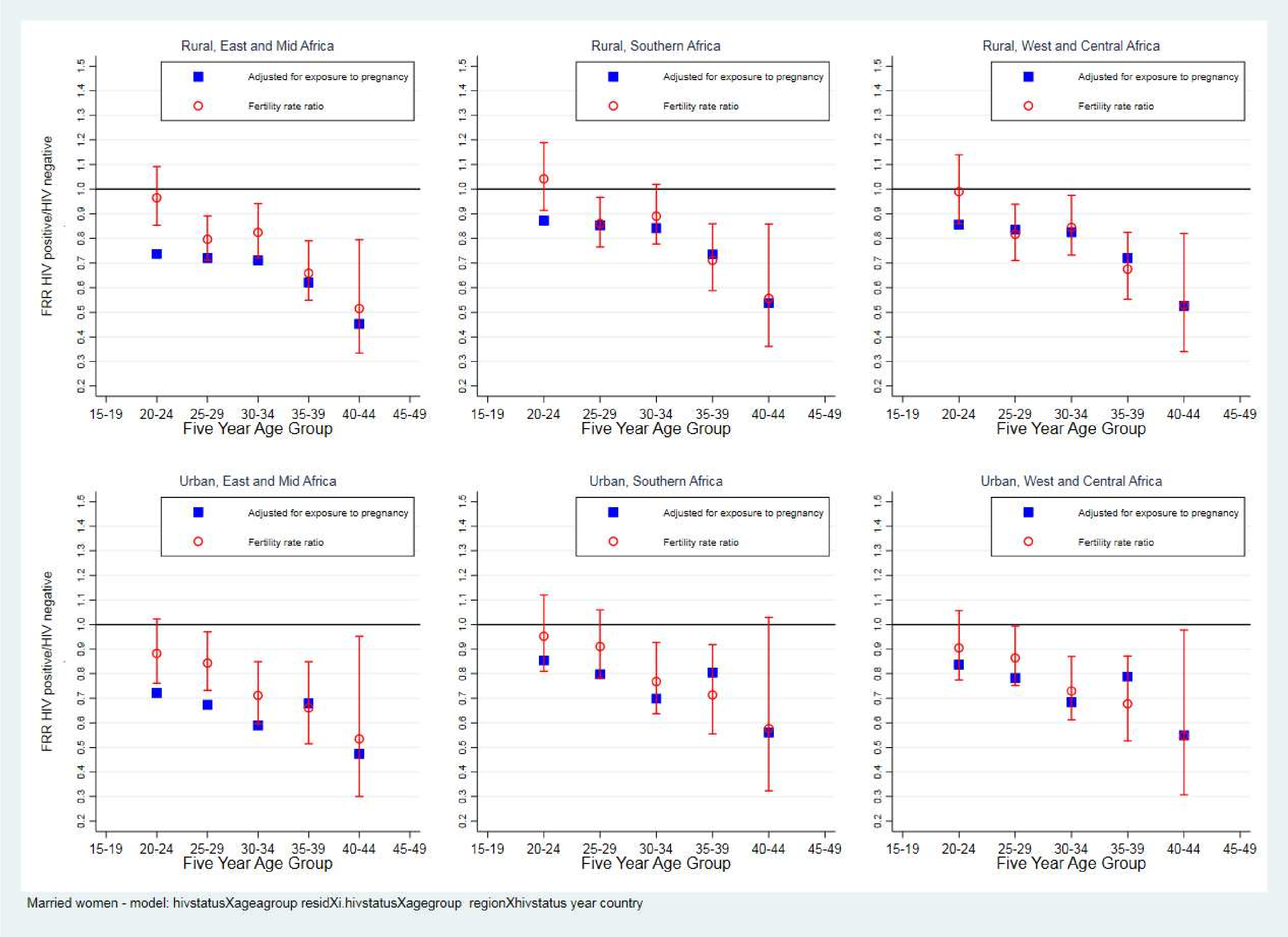

Figure M: Comparing fertility rate ratios adjusted for exposure to pregnancy for all women to married women

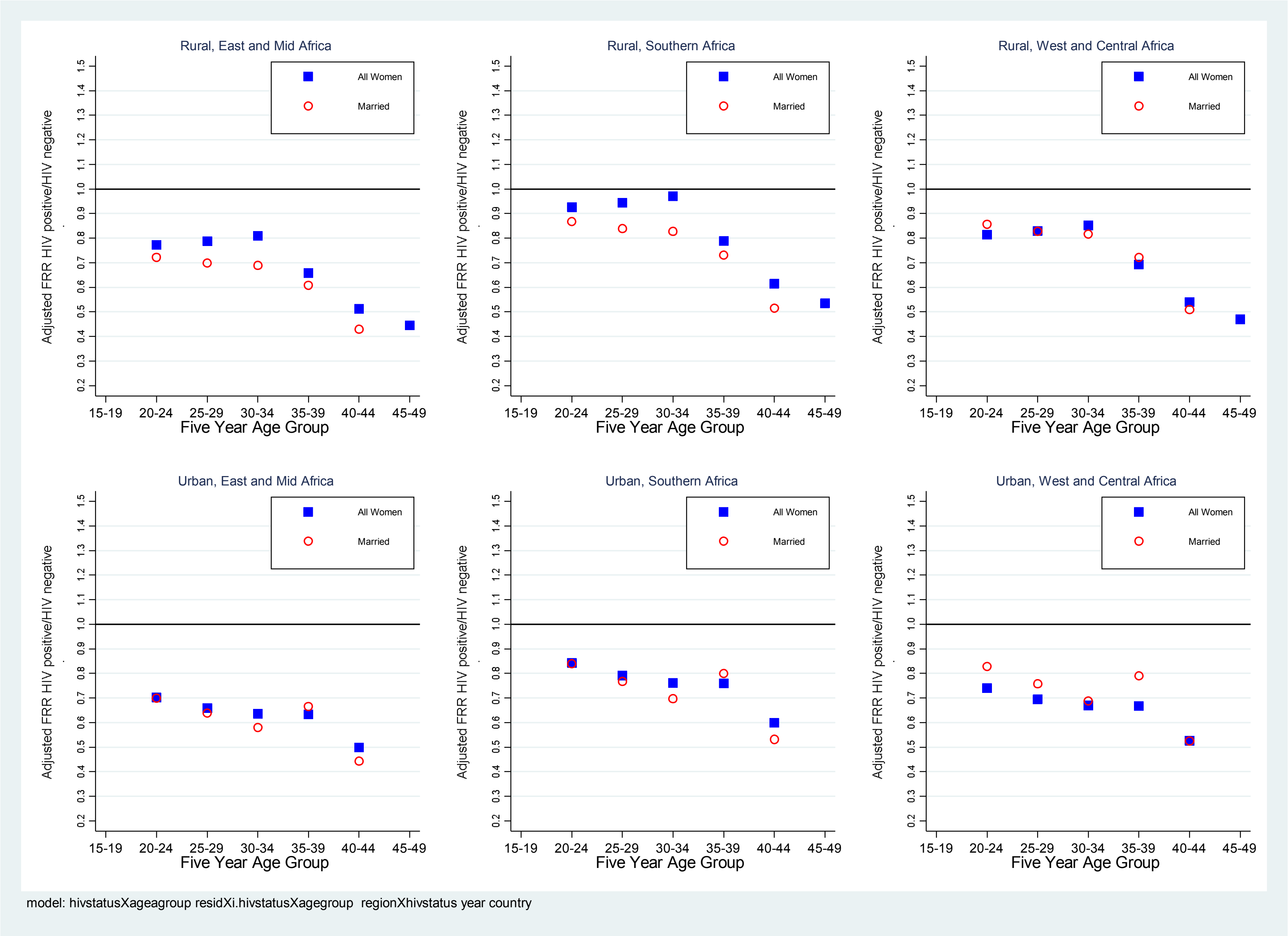

Supplement: S1 Appendix — (PDF) [file pone.0204584.s001.pdf]
